# A hybrid binary dwarf mongoose optimization algorithm with simulated annealing for feature selection on high dimensional multi-class datasets

**DOI:** 10.1038/s41598-022-18993-0

**Published:** 2022-09-02

**Authors:** Olatunji A. Akinola, Absalom E. Ezugwu, Olaide N. Oyelade, Jeffrey O. Agushaka

**Affiliations:** School of Mathematics, Statistics, and Computer Science, University of KwaZulu-Natal, King Edward Avenue, Pietermaritzburg Campus, Pietermaritzburg, 3201 KwaZulu-Natal South Africa

**Keywords:** Mathematics and computing, Computer science

## Abstract

The dwarf mongoose optimization (DMO) algorithm developed in 2022 was applied to solve continuous mechanical engineering design problems with a considerable balance of the exploration and exploitation phases as a metaheuristic approach. Still, the DMO is restricted in its exploitation phase, somewhat hindering the algorithm's optimal performance. In this paper, we proposed a new hybrid method called the BDMSAO, which combines the binary variants of the DMO (or BDMO) and simulated annealing (SA) algorithm. In the modelling and implementation of the hybrid BDMSAO algorithm, the BDMO is employed and used as the global search method and the simulated annealing (SA) as the local search component to enhance the limited exploitative mechanism of the BDMO. The new hybrid algorithm was evaluated using eighteen (18) UCI machine learning datasets of low and medium dimensions. The BDMSAO was also tested using three high-dimensional medical datasets to assess its robustness. The results showed the efficacy of the BDMSAO in solving challenging feature selection problems on varying datasets dimensions and its outperformance over ten other methods in the study. Specifically, the BDMSAO achieved an overall result of 61.11% in producing the highest classification accuracy possible and getting 100% accuracy on 9 of 18 datasets. It also yielded the maximum accuracy obtainable on the three high-dimensional datasets utilized while achieving competitive performance regarding the number of features selected.

## Introduction

Technological advancement in various fields of endeavor has resulted in a large amount of data being generated in the information industry. The massive data available today can only be meaningful if there are corresponding tools that can transform these data into information without stress. Data mining and machine learning are potent tools in this regard because there has been a tremendous growth in the use of these tools to transform massive data into meaningful information. However, this large amount of data comes with redundancies, noise, and many features which may hinder knowledge discovery activities like a classifier's performance.

Knowledge discovery (KD) activities consist of repeatedly performing data cleaning, dimensional reduction, data integration and transformation, and many other activities. These activities form part of the pre-processing tasks, without which the performance of data mining and machine learning algorithms would be significantly affected. Data is so important nowadays that it is regarded as the 'new currency.' Careful handling of the 'new currency' is required, making data mining and machine learning a fast-growing field. The dimension of the vast data available is of great concern for data miners because it impacts their ability to transform the data into meaningful information. Many data mining and machine learning tools require considerable time to carry out their tasks. Therefore, noisy data with redundant features would increase these algorithms' time complexity. For this problem to be resolved, the pre-processing step of feature selection becomes crucial, which can impact the performance of learning algorithms. The feature selection then plays a notable role in many research areas^1,2^.

Feature selection has become a prominent approach employed to remove irrelevant and unnecessary features, reducing the attributes that do not aid the purpose of classification but add more burden on computational cost and requirement for space. This process is often categorized into wrapper and filter approaches. The first employs one or more learning algorithms to extract a relevant subset of features. At the same time, the latter is independent of a learning algorithm and uses information gain, mutual information, Laplacian score, and many more to select useful features^3,4^. Generally, the wrapper methods yield better results in terms of classification accuracy^5^ than the filter methods, which produce results faster than the wrapper approach. However, the wrapper methods are computationally expensive. This wrapper feature selection method is an optimization problem^6,7^. In tackling this optimization problem of feature selection, metaheuristic algorithms have played a prominent role in recent years. Among these methods are work done^6–11^ which produced better solutions in comparison with the exact techniques like recursive feature elimination, mutual information, Laplacian score.

The feature selection problem aims to locate the most appropriate or best subset of features, thereby choosing the minimum features possible and maximizing the classification accuracy. This has become a daunting task due to its contradicting, multi-objective nature. In recent years, metaheuristic algorithms have been utilized to solve this issue. As a result of the increasing time required to find the best feature subsets, especially in a high-dimension dataset, feature selection is considered an NP-hard problem^12^. Considering a dataset with an N feature, a sum of 2^N − 1 features combination is needed to investigate the location of the optima feature^13,14^. Therefore, a high-performing metaheuristic algorithm is required to reduce the processing time posed by this kind of problem. The random forest will likely solve this problem,however, the trees tend not to go deep and have a high bias^15^.

Metaheuristic algorithms are deemed more appropriate for solving such problems due to their ability to cater to the worst-case scenario^16^. Many of these metaheuristic algorithms are found in literature, some of which include Particle Swarm Optimization (PSO)^17^, Genetic Algorithm (GA)^18^, Whale Optimization Algorithm (WOA)^19^, DragonFly Algorithm (DA)^20^, Cuckoo Search Optimization Algorithm (CSO)^21^, Harmony Search algorithm (HS)^22^, Grey Wolf Optimizer (GWO)^23^, Krill Herd Algorithm (KHA)^24^, Prairie Dog Optimization Algorithm (PDO)^25^, and more. Each of these optimizers has been used to solve different problems with varying successes. Generally, metaheuristic algorithms' search process relies on the equilibrium between the diversification and intensification phases. Diversification is also an exploration where candidate solutions that are not neighboring are evaluated. Meanwhile, intensification is also regarded as exploitation where neighboring solutions are intensively searched for the best solutions. The importance of these two phases cannot be overemphasized as a factor in finding the best solution(s).

Several researchers have employed metaheuristic algorithms to solve many feature selection problems^6,7,26^. Due to the great demand for these algorithms, researchers have invested a lot of time and effort in developing superior algorithms capable of generating high-quality solutions for the candidate problem. Since metaheuristics approaches can only give near-optimal results, developing new methods or improving existing algorithms to obtain better results or optimal solutions has become an ongoing endeavor. Furthermore, the No Free Lunch (NFL) theorem assumes that no single algorithm can produce optimal results for all optimization problems^27^. We can therefore conclude that there is no universal or one-size-fits-all algorithm for every optimization problem that can produce the best results. The reference to this theory has motivated research in this area. More researchers are developing efficient and novel metaheuristic algorithms to solve the FS problems.

Though many metaheuristic algorithms have been used to solve the feature selection problems in the literature, most have inherent drawbacks or shortcomings^28^. Typically, these algorithms' performance largely depends on the innate traits of the datasets used to train those models. Usually, hybrid algorithms are often considered a better preference because the shortfall of one method is enhanced by the strength of the other^29^. Therefore, the success of the hybrid implementation of FS-based metaheuristic algorithms has motivated the current study, where a hybrid algorithm is proposed by hybridizing the binary variant of the standard DMO algorithm with the simulated annealing (SA). In this study, the binary DMO (or BDMO) was enhanced by improving its capacity to adequately exploit the intensification phase of the underlying problem landscape to produce better solutions. Therefore, the focus is on implementing a new hybrid solution that uses simulated annealing (SA)^30^ to augment the identified limitation of the binary dwarf mongoose optimizer. The hybrid is subsequently employed to solve the problems of high-dimensional feature selection datasets. The new hybrid algorithm is called the binary dwarf mongoose simulated annealing optimization (BDMSAO) algorithm.

In the new proposed feature selection method, the SA algorithm is used as a component in the BDMO or as a low-level team player in conjunction with the BDMO algorithm to increase the quality of the final feature selection results. Precisely, the SA is used to search the neighborhood of the best search agent to ensure that the local optima are enhanced. In addition, the SA is also employed after the BDMO is done exploring the solution search space to enhance the best-found solution, which afterwards is identified as the global best solution. It is noteworthy that several researchers have recently proposed similar hybrid methods, including the work presented in^1,31,32^. The results obtained by these hybrid methods proved their efficiency and outperformance over other related state-of-the-art techniques. Thus, in addition, this certainly also motivated our proposed hybrid version of the DMO. The major contribution of this study can be summarized as follows:

- A hybrid FS method called BDMSAO is introduced using BDMO and high-performing simulated annealing (SA).
- The proposed BDMSAO method is evaluated using 18 standard UCI datasets using the K-nearest Neighbors (KNN) classifier to prove its effectiveness.
- Also, the BDMSAO is applied to solve three high-dimensional datasets from the Arizona State University FS repository to prove its robustness further
- The performance of the proposed FS method is compared with many state-of-the-art metaheuristic-based FS methods.

The remaining part of this paper is structured as follows: “Related work” section reviews related work. “Preliminaries” presents the two methods, BDMO and SA, that were hybridized in this study. Similarly, “The proposed hybrid method” presents the proposed method considering its solution presentation, fitness function, and computational complexity. In “Experimental results and discussion”, the experimental results of this study are discussed, and the statistical analysis test results are presented. “Testing on high-dimensional datasets” discusses the use of the proposed method on high-dimensional datasets to show its robustness. Finally, “Conclusion and future work” concludes this work by giving its limitation and future direction.

## Related work

Recently, metaheuristic algorithms have gained ground in solving optimization problems, and these methods are regularly undergoing enormous improvements from researchers. Metaheuristic algorithms have become pivotal in finding optimal solutions through algorithms' learning using the process of iteration. In^33^, metaheuristic algorithms were divided into population-based and single solution-based. Also^34^, categorized these algorithms into non-nature inspired and nature-inspired metaheuristics. Many researchers have developed several hybrid forms of metaheuristic algorithms to solve feature selection problems. The hybridized methods have proven their superior performance in solving practical and real-world problems^29^. In the first hybrid method (metaheuristic), the Genetic Algorithm (GA) was combined with a local search algorithm to solve the optimization problem of feature selection^35^. From the inspirational viewpoint, metaheuristic algorithms can be generally grouped into swarm-based, physics-based, evolutionary-based, and human-based.

### Swarm-based algorithms

Algorithms in this group are inspired by the social interaction or behaviour of birds, animals, insects, fish, schools, herds and so on. The main underlying idea of these algorithms is that everyone has a particular behaviour but coming together as a group or team and harnessing their joint effort enables them to solve very complex optimization problems. Several algorithms have been developed in this category in the last two decades, and researchers have also developed variants of some popular ones. Others have or/are still hybridizing them to solve various optimization problems. One of the prominent ones is the PSO^17^ which has gained so much attention due to its rich mathematical basis for solving problems. Other algorithms in this group are Cuckoo Search (CS)^21^, Grey Wolf Optimizer (GWO) by^23^, Krill Herd Algorithm (KH)^24^, Whale Optimization Algorithm (WOA)^36^, Dwarf Mongoose Optimization (DMO)^37^ algorithm, Gazelle Optimization Algorithm (GOA)^38^ etc.

As one of the most notable algorithms in the swarm-based category, the PSO has also been greatly hybridized to solve the feature selection problem. In^39^, a local search algorithm was employed to assist the PSO in searching for the optimal solution and selecting the minimum reducts in relation to their correlation information. Talbi et al.^40^ proposed a wrapper-based hybrid GA-SA method called GPSO using SVM as the classifier, and the work of^41^ presented a multi-objective and hybrid mutation operator, which were both applied to the classification of microarray data. The study in^42^ presented a novel hybridization of the GA with PSO in optimizing feature sets on the datasets of Digital Mammogram. The studies in^43,44^ presented two different wrapper-based feature selection methods that hybridized the GA with Ant Colony Optimiser (ACO). Another study^45^ combined the GA and Cuckoo search algorithms as a wrapper-based method to tackle the feature selection problem. Other hybrid methods include Harmony Search Algorithm and Stochastic Local Search (Nakkaa & Boughaci, 2016), Artificial Bee Colony (ABC) and Differential Evolution (DE) algorithms^46^.

### Evolutionary-based algorithms

Algorithms that fall under this category are inspired by nature or through biological process of evolution and begin their process by randomly generating their population solutions. The foremost algorithm in this category is the Genetic Algorithm (GA)^18^ which generated its fittest individual using mutation and crossover in every generation. The GA has attracted a lot of attention with the creation of different variants, and improvements have been employed to solve many real-world problems. Other popular algorithms developed in this group include genetic programming^47^, tabu search^48^, evolution strategy, differential evolution, flower pollination algorithm^49^, memetic algorithm^50^, Biogeography-Based Optimization^51^, and more.

Apart from the presentation of these evolutional metaheuristic algorithms, the GA being the prominent algorithm in the evolutionary category has attracted significant attention where it was hybridized with other methods to solve different optimization problems^52–55^ where those studies revealed the potency (in terms) of producing better output in comparison with either other local or global search models. It has also been widely hybridized in the domain of feature selection. The GA was also combined with the SA as a filter approach^56^ to enhance the GA's local search capability to solve the feature selection problem. This method was evaluated using eight datasets from the UCI machine learning repository. It performed better in selecting the minimum number of a subset of features than other popular methods. Also in^57^, the authors proposed a memetic feature selection algorithm where the study utilized the fuzzy logic in controlling the major parameters on two local search techniques, which was later combined with the GA. In application to the wrapper-based method, the crossover operator of the GA was combined into the metropolis acceptance criterium of the SA^58^. Furthermore, it was hybridized in^59^ in classifying power disturbance in the problem of Power Quality (PQ) which also optimize SVM parameters. Moreso in^60^, it was combined with Tabu Search, which employed the Fuzzy ARTMAP Neural Network to evaluate the wrapper feature selection method.

### Physics-based algorithms

These algorithms draw their inspiration from the laws of physics in the world. Physics-based methods are inspired by physics principles, chemistry, music, complex dynamic systems, physics and metallurgy to mathematics^1^. Some prominent algorithms in this group include Gravitational Search Algorithms^61^, Atom Search Optimizer^62^, Ray Optimization^63^, Galaxy-Base Search Algorithm^64^, Equilibrium Optimizer (EO)^65^, Sine Cosine Algorithm (CSA)^20^ and so on.

In^66^, a hybrid metaheuristic feature selection method was proposed using a Golden Ratio Optimization (GRO) and Equilibrium Optimization (EO) algorithms called the Golden Ratio based Equilibrium Optimization (GREO) algorithm, which was applied in speech emotion recognition. Also, the study conducted in^67^ presented a hybrid feature selection method that is based on the ReliefF filter technique and EO known as RBEO-LS, which have two phases: the first employed the ReliefR algorithm at the pre-processing stage for feature weights assignment, and the second utilized binary EO (BEO) as a wrapper search technique.

### Human-based algorithms

Algorithms in this category are inspired by activities performed by humans or human behaviours. Human beings are involved in various activities that affect their performance, and researchers use these behaviours to develop algorithms. The most prevalent algorithms here are Teaching Learning-Based Optimization (TLBO) by^68^ and League Championship algorithms (LCA)^69^. Others include Exchange Market Algorithm (EMA) by (Ghorbani & Babaei, ^70^), Social-Based Algorithm (SBA) by^71^, Seeker Optimization Algorithm (SOA) by^72^, etc. It is observed from the literature that not many algorithms are human inspired. TLBO, a well-known method in this category, was hybridized in^73^ with extreme learning machines (ELM), referred to as TLBO-ELM in solving data classification problems which feature selection falls under. It was tested on some UCI benchmark datasets.

With the strength of metaheuristic algorithms comes to some issues, among which is premature convergence that results in locating limited optimal solutions. Frequently, researchers combine these algorithms with other methods like local search techniques. Generally, the local search algorithm tries to conduct an intensive search of each region of the solution, which can outperform existing metaheuristic solutions. Among the existing local search methods are Simulated Annealing (SA)^30^, tabu search^48^, and Hill climbing (HC). The HC have variations like the βHC, Adaptive βHC and Late Acceptance HC (LAHC). Several works exist in the literature that employed the combination of local search strategy with metaheuristic algorithms. Some of them includes^1,74–78^.

### Preliminaries

#### Dwarf Mongoose Optimization Algorithm

DMO^37^ is a population-based stochastic metaheuristic algorithm inspired by the foraging and social behaviour of dwarf mongoose, also called Helogale. Each dwarf mongoose search for food individually since food search is not a collective exercise, but foraging is done collectively. Due to the seminomadic attribute of these animals, the building of a sleeping mound is close to an abundant source of food. The algorithm mathematically models the lifestyle of this animal to solve optimization problems.

All population-based optimization algorithms commence with random initialization. After that, because of the intensification and diversification rules, every solution gathers around the global best optima. Similarly, the DMO starts its solution by initializing the mongoose's candidate population. This population is generated stochastically between a particular problem's lower and upper bounds.

1 $$X = \left[ {\begin{array}{*{20}c} {\begin{array}{*{20}c} {x_{1,1} } & {\begin{array}{*{20}c} {x_{1,2} } & \cdots \\ \end{array} } & {\begin{array}{*{20}c} {x_{1,d - 1} } & {x_{1,d} } \\ \end{array} } \\ \end{array} } \\ {\begin{array}{*{20}c} {x_{2,1} } & {\begin{array}{*{20}c} {x_{2,2} } & \cdots \\ \end{array} } & {\begin{array}{*{20}c} {x_{2,d - 1} } & {x_{2,d} } \\ \end{array} } \\ \end{array} } \\ {\begin{array}{*{20}c} {\begin{array}{*{20}c} \vdots & { \begin{array}{*{20}c} \vdots & {x_{i,j} } \\ \end{array} } & { \begin{array}{*{20}c} \vdots & { \vdots } \\ \end{array} } \\ \end{array} } \\ {\begin{array}{*{20}c} {x_{n,1} } & {\begin{array}{*{20}c} {x_{n,2} } & \cdots \\ \end{array} } & {\begin{array}{*{20}c} {x_{n,d - 1} } & {x_{n,d} } \\ \end{array} } \\ \end{array} } \\ \end{array} } \\ \end{array} } \right]$$

where X represents the set of the candidates' present population that are generated randomly using Eq. (2), $x_{i,j}$ indicates the position of the jth dimension of the ith population, n indicates the population size, and d is the dimension of the problem.

2 $$x_{i,j} = unifrnd\left( {VarMin,VarMax,VarSize} \right){ }$$

where $unifrnd$ is a random number that is distributed uniformly, $VarMin{ }and{ }VarMax$ are lower bound and upper bound, respectively $VarSize$ is the dimension of the problem. The best solution at each iteration is the best solution obtained so far.

Like every metaheuristic algorithm, there are two phases in the DMO: exploitation (individual mongoose carries out a thorough search in each search space), also called intensification and exploration (a random search for a new abundant food source or new sleeping mound) or diversification. Three major social structures of the DMO carry out the activities of the two phases mentioned: the alpha group, scout group, and babysitters.

The alpha female $\left( \alpha \right){ }$ is the family unit controller and is selected using Eq. (3).

3 $$\alpha = \frac{{fit_{i} }}{{\mathop \sum \nolimits_{i = 1}^{n} fit_{i} }}$$

$n - bs$ matches the number of mongooses in the alpha group. The number of babysitters is denoted by $bs,$ and $peep$ represents the sound of female alpha to the path of the other unit members.

The sleeping mound is determined by abundant food which is expressed in Eq. (4).

4 $$X_{i + 1} = X_{i} + phi{*}peep$$

where $phi$ is a random uniformly distributed number [− 1,1], after each iteration, there is an evaluation of the sleeping mound; Eq. (5) represents this.

5 $$sm_{i} = \frac{{fit_{i + 1} - fit_{i} }}{{{\text{max}}\left\{ {\left| {fit_{i + 1} ,fit_{i} } \right|} \right\}}}$$

when a sleeping mound is found, an average value is derived using Eq. (6)

6 $$\varphi = \frac{{\mathop \sum \nolimits_{i = 1}^{n} sm_{i} }}{n}$$

Once the babysitter exchange criterium is attained, the next phase is the scouting, which evaluates the next sleeping mound determined by another food source.

Since mongoose is known not to return to a prior sleeping mound, the scout group goes searching for the next sleeping mound to ensure exploration. The mongoose is known to forage and scout simultaneously in DMO with the justification that the farther the unit forage, the likelihood of finding the next sleeping mound. This is simulated using Eq. (7).

7 $$X_{i + 1} = \left\{ {\begin{array}{*{20}c} {X_{i} - CF*phi*rand*[X_{i} - \vec{M}]} & {if\; \varphi_{i + 1} > \varphi_{i} } \\ {X_{i} + CF*phi*rand*[X_{i} - \vec{M}]} & {else} \\ \end{array} } \right.$$

where ${ }rand{\text{ is a random number between }}\left[ {0,1} \right],$ $CF = \left( {1 - \frac{iter}{{Max_{iter} }}} \right)^{{\left( {2\frac{iter}{{Max_{iter} }}} \right)}}$ shows the parameter that directs the collective-volatile movement of the mongoose's group that linearly decreases during iterations. $\vec{M} = \mathop \sum \limits_{i = 1}^{n} \frac{{X_{i} \times sm_{i} }}{{X_{i} }}$ connotes the vector that motivates the movement of mongoose to a new sleeping mound.

The babysitter's group remains with the juveniles when the scouting and foraging group searches for a sleeping mound and food source. The number of the members of this group is removed from the total number of candidate population as they do not go to forage or scout until the babysitter exchange parameter is met in Eq. (7). In the following section, the proposed binary variant of DMO is presented with the hybrid method of simulated annealing (SA).

## The proposed hybrid method

In this section, the representation of the solution, the fitness function utilized and the proposed method's computation complexity are elaborated. The feature selection problem, an optimization problem represented in binary form, and its solution confined to 0 s and 1 s were taken care of by the BDMO. The agents update their position in each iteration using the BDMO optimization rules and afterwards pass the solution to the SA to locate the better neighborhood solution to improve and refine the results. As a multi-objective optimization problem where two opposing objectives of high classification accuracy and minimal features selected as possible need to be met. The achievement of these two objectives determines how best a solution is. In the proposed method, the BDMO utilizes the tournament selection mechanism to advance the algorithm's diversification capability, which affords a high chance of selecting weak solutions while searching for promising ones.

The DMO is a recent metaheuristic algorithm which proves its efficacy in solving mechanical optimization problems. The algorithm employs a Tau operator, which signifies that if a new food source is not found mindless, the fitness value of the present solution and the one being operated upon the intensification should be performed (Eq. 7). This operator was replaced with the SA as a local search technique that takes an initial state of a solution, processes it, and replaces the improved solution in place of the original one. This technique represents the hybridization of the BDMO and SA as a local search method.

### Solution representation

The feature selection problem being an optimization problem has its output $\in \left[ {0,1} \right]$. The zero indicates that the feature is redundant or irrelevant and is thereby rejected, while one signifies that the feature is useful and therefore selected. The possibility that the results might be out of range cannot be ruled out. Therefore, the binarization function is applied to every agent to ensure they remain within the specified range. This is performed using Eq. (8). To select a feature, the position index must be 0.5 and above, which rounds the value to 1, and for any feature to be rejected, its position index must be less than 0.5, which is rounded down to 0.

8 $$BestSol^{d} = \left\{ {\begin{array}{*{20}c} {BestSol_{i}^{d} > 0.5\, feature\, is\, selected} \\ {BestSol_{i}^{d} \le 0.5\, unselected\, feature } \\ \end{array} } \right.$$

where $BestSol_{i}^{d}$ is the best solution $i$ in dimension $d$. Thereby, a mongoose's position shows that a feature set is selected as the value of position increases for the dimensions^79^.

### Fitness function

Selecting a useful feature that assists the classifier in recognizing a class of a sample in a dataset is challenging. During the selection process of relevant features, there is a need to remove the redundant ones for the sake of classification automatically and to maximize the accuracy of the classification problem when the feature selected is to be used^80^. In this work, the BDMSAO is utilized to locate the best feature subset and employ the KNN classifier to calculate the classification accuracy. The classification accuracy of this model $A_{c}$ is gotten by a classifier, $b_{s}$ represent the feature subset dimension, and the total number of attributes contained in the dataset is signified by $D_{t}$. Therefore, the classification error is $1 - A_{c}$ and the subset of selected features from the dataset is denoted by $\frac{{d_{s} }}{{D_{t} }}$. Hence, the fitness function is defined as:

9 $$\downarrow Fitness = \mu \cdot \left( {1 - A_{c} } \right) + (1 - \mu ) \cdot \frac{{d_{s} }}{{D_{t} }}$$

where $\mu$ $\in$ [0,1] is the weight assigned to the error classification.

### The BDMSAO algorithm

The binary version of DMO is proposed in this study to solve the problem of feature selection for many benchmark datasets. The aim is to investigate the performance of the new hybrid algorithm in solving the challenging problem of selecting minimal features from high-density datasets. The resulting BDMO algorithm is supported by the SA method to boost its operations in the local search tasks. The evaluation of the fitness function in the proposed BDMO method utilizes Eqs. (10–12).

10 $$xf_{i} = size\left( {flat\left( {x_{i} } \right)} \right)$$

11 $$xs_{i} = \left( {1 - 0.99} \right)* \frac{{sum\left( {x_{i} } \right)}}{{xf_{i} }}$$

12 $$f_{i} = 0.99*(1 - classifier\left( {X\left[ {:xf} \right], Y\left[ {:xf} \right] } \right) + xs_{i}$$

where the $xf_{i}$ represents the number of items in a normalized value of $x_{i}$, and $xs_{i}$ is applied to compute the actual fitness value of $x_{i}$ which is assigned to $f_{i}$.


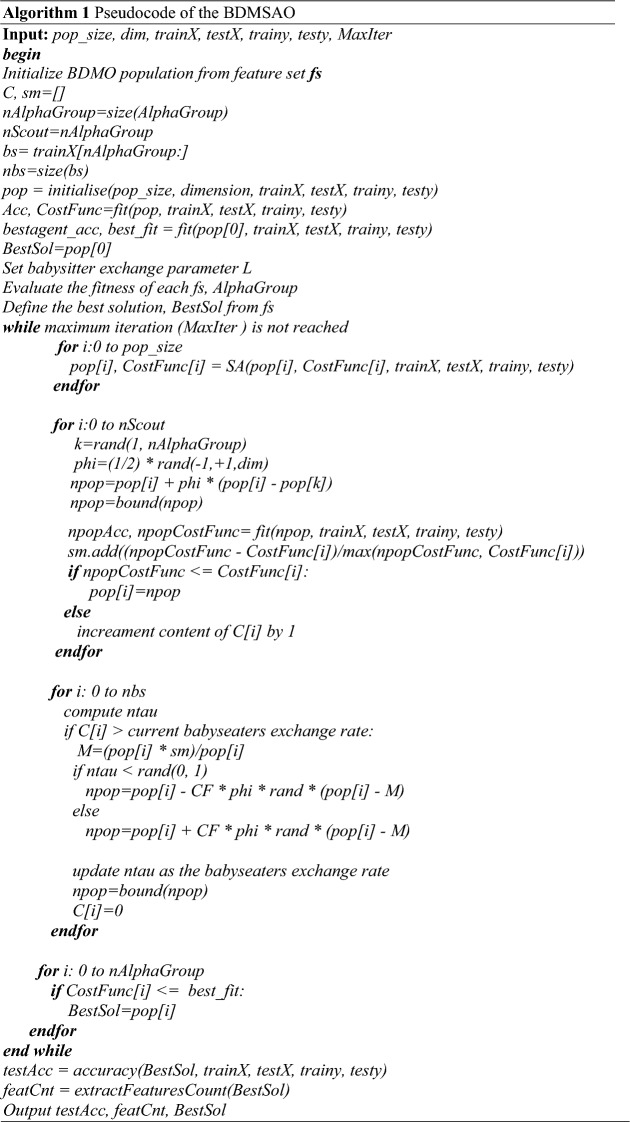


Algorithm 1 is a detailed listing of the pseudocode for the proposed hybrid algorithm BDMSAO. The search mechanism of the alpha groups in the DMO is now replaced by the SA for an improved local search operation. The algorithm accepts datasets prepared as *trainX, testX, trainy, testy,* and in addition the population size, the number of iterations, and the dimension of population. Using these inputs, the population is initialized and computation for the fitness value for each individual in the population is derived. During the iteration, three search processes are evaluated including the SA-based search, scout group-based search and the baby seater-based search. These three-level searches apply nature-specific operations which demonstrates the balance for the exploration and exploitation phases of the proposed BDMSAO algorithm. Specifically, the SA is adapted to improve the local search of BDMO for improved performance. Once the three stages of the search process are completed, the global best solution is identified so that classification accuracy for the solution is computed using the datasets. The algorithm returns the computed number of features selected, the accuracy of the classification leading to the selected number of features, and the best solution.

### Complexity of computation

The computation complexity of every metaheuristic algorithm depends on the time each candidate takes to update its positions, the maximum iteration value, other operations such as sorting or comparison, and variable update time. The computational complexity of the BDMSAO is $O\left( {Max_{iter} *Pop_{size} *Dim_{s} *T_{fitness} } \right), where$ $Max_{iter}$ is the maximum iteration number, $Pop_{size}$ is the population size, $Dim_{s}$ denotes the search space dimension, $T_{fitness}$ represent the classifier's time required to calculate the fitness of a given solution. The SA is employed to locate the best solutions if they can be found in the neighborhood of the present solution. In terms of $O - notation$, the SA does not significantly affect the computation cost.

**Figure 1:**
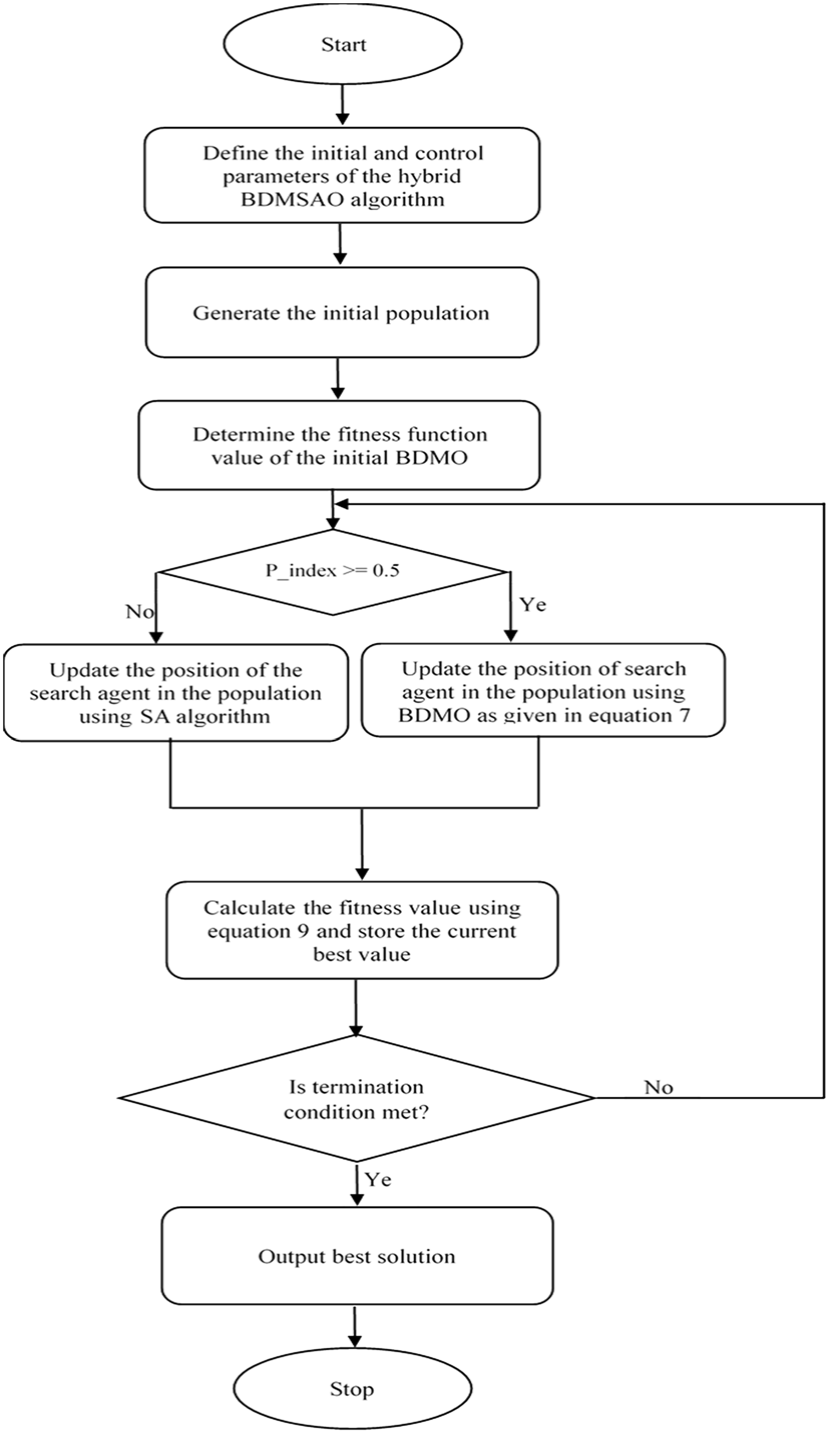
Flowchart illustrating the algorithmic structure of the BDMSAO algorithm.

The optimization steps of the developed hybrid BDMSAO algorithm for solving feature selection problems is presented in Fig. 1. In the given figure, the hybrid BDMSAO's first step is defining all the parameters (which includes both BDMO and SA algorithms parameters, respectively). Then the next step is to generate the population representing a set of solutions for the feature selection problem. Subsequently, the fitness function of the individual candidate solution is determined based on evaluating and selecting the best features, after which the current best solution is identified and retained. The next step for the BDMSAO algorithm is to update the current population by using either the BDMO or SA algorithms, again depending on the quality of the fitness function. The process is such that if the probability of fitness function for the current solution is greater than 0.5, then the BDMO is selected for update. Otherwise, the SA algorithm is used to update the current population. Note that the probability above is computed as a factor of the position index (P_index) being $> = 0.5$. Thereafter, the fitness function for each solution is computed using Eq. (9), and the best solution is determined after updating the population. The next step is for the BDMSAO to check if the stopping criteria have been met and if yes, then the algorithm returns the overall candidate's best solution. Otherwise, the algorithm will iteratively repeat the previous steps from checking whether P_index is $> = 0.5$ until finally the stop condition is reached.

## Experimental results and discussion

### Dataset (low, medium and high-dimensional)

To evaluate the performance of the BDMSAO, eighteen University of California Irvine (UCI) low, medium, & high-dimensional datasets and two high-dimensional datasets from the Arizona State University feature selection repository. The details of the datasets used, including their feature number (N), instances, classes, and categories, are presented in Table 1. The high-dimensional datasets contain numerous features of at least two thousand (2000), and few of the datasets are multi-class in nature, ranging from 3 to 9 classes. These high-dimensional datasets usually represent real-world scenarios and are therefore more challenging. This allows us to ascertain the robustness of the proposed feature selection method. Although, there are studies in the literature where some feature selection methods were utilized to solve high-dimensional datasets problems, one of which is the work presented in^81^. However, the maximum number of features in those datasets was only limited to N = 4703 compared to the more high-dimensional feature sizes utilized in the current study. Not many metaheuristic algorithms perform reasonably on high-dimensional and multi-class datasets.

**Table 1:** Datasets and their properties.

Number	Datasets	# features	# instances	# Classes	Categories
1	Breastcancer	9	699	2	Biological
2	BreastEW	30	569	2	Biological
3	CongressEW	16	435	2	Political
4	Exactly	13	1000	2	Biological
5	Exactly2	13	1000	2	Biological
6	HeartEW	13	270	2	Biological
7	IonosphereEW	18	351	4	Electromagnetic
8	KrvskpEW	36	3196	2	Game
9	Lymphography	18	148	4	Biological
10	M-of-n	13	1000	2	Biological
11	PengiunEW	325	73	2	Biological
12	SonarEW	60	205	2	Biological
13	SpectEW	22	267	2	Biological
14	Tic-tac-toe	9	958	2	Game
15	Vote	16	300	2	Political
16	WaveformEW	40	5000	3	Physical
17	WineEW	13	178	3	Chemical
18	Zoo	16	101	6	Artificial

### Experimental setup

**Figure 2:**
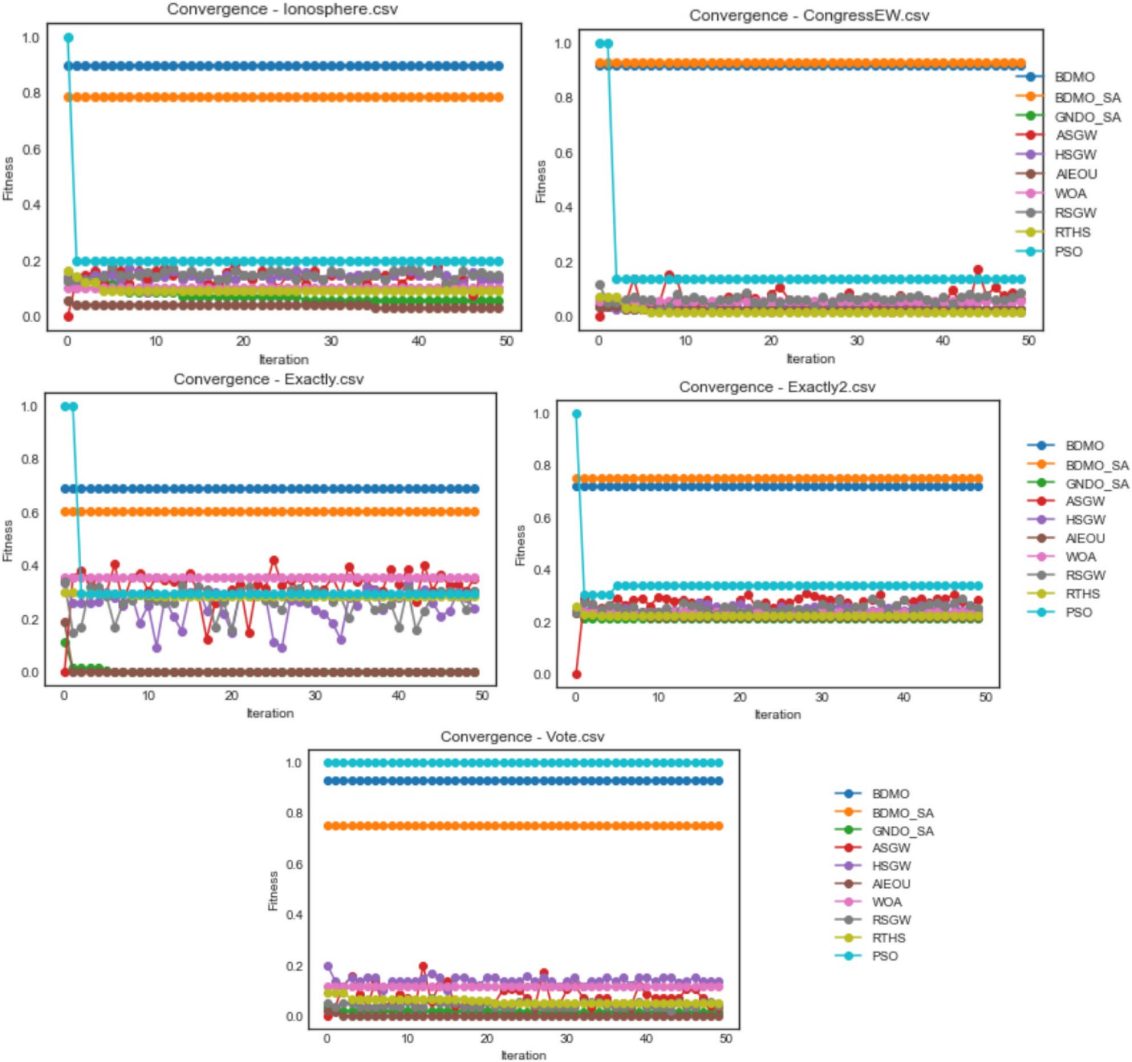
Convergence behavior of all algorithms on the ionosphere, congressEW, Exactly, Exactly2, and Vote datasets.

The proposed BDMSAO was implemented using python. Most often, parameters play a key role in determining the outcome of multi-agent algorithms, particularly the agents' number and iteration's total number, which heavily influence the algorithm's performance. Therefore, the experiment was performed considering different population sizes to determine the suitable size of the population and number of iterations. To test the efficiency of this hybrid approach, we compared the proposed BDMSOA with the BDMO. The classification accuracy and number of features selected are shown in Tables 3 and 5 using various population sizes from 10 to 50. The convergence graphs for both methods are also depicted in Fig. 2 to show the solution's optimal position over the total iteration number of 50. For a fair comparison, each dataset was run 10 times, and the average values of the runs were taken. The computer configuration for this implementation is Core i7, 3.60 GHz CPU with 16 GB RAM. The finding of this experiment reveals that the population size of 10 produced better results which will serve as the basis for comparison in this study. Table 2 presents the parameter setting for the developed hybrid FS methods.

**Table 2:** Experimental parameter setting.

Parameter	Value
K-fold cross-validation number	10
Agent number	10–0
Number of runs	10
Maximum iterations	50
Dimension of problems	Number of available features in the dataset N
Peep (vocalization) of female alpha mongoose in BDMO	Controlled
Babysitters in BDMO	Controlled
initial temperature	2 ∗|N|
Cooling schedule (T)	0.93

### Result and discussion

This subsection discusses the results generated by BDMSAO and BDMO, which were evaluated using eighteen datasets from the UCI repository, with details in Table 1. Since the proposed method is a wrapper-based approach, the utilized classifier is K-Near Neighbor (KNN) since it is a well-known and most widely used classifier in wrapper-based feature selection^82^ and it was used with $K = 5$ in the experiment. The generated results show the outperformance of the BDMSAO over the binary DMO. The outcome of the experiment in Tables 3–4 indicates the efficacy of the proposed hybrid method over the BDMO in locating better solutions. We can conclude that the BDMSAO performed better on UCI datasets than the BDMO.

**Table 3:** The classification accuracy of BDMO and BDMSAO on different population sizes.

Agent_size	10		20		30		40		50	
Dataset	BDMO	BDMSAO	BDMO	BDMSAO	BDMO	BDMSAO	BDMO	BDMSAO	BDMO	BDMSAO
Breastcancer	93.57	100	69.29	99.29	92.86	100	65.71	99.29	90.71	99.29
BreastEW	89.47	98.25	92.11	96.49	91.23	98.25	92.11	98.25	86.84	98.25
CongressEW	94.25	98.85	91.95	100	87.36	98.85	85.06	100	97.70	100
Exactly	65.50	100	63	100	66	100	66	100	67.5	100
Exactly2	70	79	74	79.50	73	79.50	73	80	67	80.50
HeartEW	79.63	90.74	73.33	92.59	64.81	92.59	62.96	96.30	68.52	90.74
IonosphereEW	88.57	97.14	82.86	97.14	84.29	95.71	82.86	95.71	82.86	98.57
KrvskpEW	66.67	98.75	77.15	98.44	100	98.75	60.72	98.75	72.30	98.59
Lymphography	73.33	100	76.67	96.67	70	93.33	63.33	100	76.67	96.67
M-of-n	68	100	67	100	63	100	67	100	67.5	100
PengiunEW	73.33	100	80	100	80	100	73.33	100	80	93.33
SonarEW	71.19	100	73.81	97.62	71.42	100	64.29	97.62	76.19	97.62
SpectEW	76.19	97.62	64.29	97.62	66.67	100	61.90	95.24	80.95	97.62
Tic-tac-toe	65.10	84.38	65.10	82.81	61.98	83.85	62.50	84.90	64.06	86.46
Vote	88.33	100	86.67	100	93.33	100	88.33	100	80	100
WaveformEW	66.30	85.20	62.80	85	67.10	84.60	60	85.50	69.20	86
Wine	88.89	100	83.33	100	86.11	100	94.44	100	86.11	100
Zoo	90	100	90	100	90	100	85	100	85	100

**Table 4:** Number of features selected by BDMO and BDMSAO.

Agent_No dataset	10		20		30		40		50	
	BDMO	BDMSAO	BDMO	BDMSAO	BDMO	BDMSAO	BDMO	BDMSAO	BDMO	BDMSAO
Breastcancer	2	4	1	3	2	4	4	3	1	4
BreastEW	14	7	4	5	14	12	2	12	4	10
CongressEW	6	3	2.03	4	5.85	4	2.52	4	6.71	4
Exactly	6	6	4	6	3.66	6	6	6	6	6
Exactly2	3	6	2	8	5	7	5	8	2	9
HeartEW	4.74	4	4.92	5	5.92	2	6	3	6.30	4
IonosphereEW	14	19	6	11	5	11	1	9	9	9
KrvskpEW	17	19	4.16	18	0	24	4.83	18	13.45	20
Lymphography	6	10	5.34	6	4.65	3	2.8	10	3.54	5
M-of-n	6	6	2	6	1	6	2	6	5	6
PengiunEW	69	132	152	162	177	135	13	77	72	108
SonarEW	16	26	7	20	5	26	8	24	16	32
SpectEW	29	22	7	29	2	21	13	22	4	25
Tic-tac-toe	1	4	2	5	3	4	2	6	1	6
Vote	1	3	2	2	7	3	5	3	2	3
WaveformEW	2	24	5	22	1	22	5	24	1	22
Wine	5.18	4	3	3	1	4	4	2	6	2
Zoo	6.21	5	8	5	8	4	6	4	7.91	4

A critical inspection of the results in Table 3 indicates that BDMSAO generates better results than BDMO on all datasets. The classification accuracy produced is greater than 90% on 16 of 18 datasets (88.88%) except on Exactly2 & Tic-tac-toe and yielded 100% on 9 of 18 datasets (50%). In the number of features selected, the BDMSAO selected fewer features on 6 datasets (BreastEW, CongressEW, HeartEW, SpectEW, Wine & Zoo), the same number of features on 2 datasets (Exactly & M-of-n), and BDMO selected fewer datasets on 11 datasets.

The convergence behavior of both methods is shown in Fig. 2. It is observed that each algorithm converges steadily in all datasets. However, the BDMSAO achieved a better convergence to show its superiority over the BDMO.

Furthermore, the fitness function's optimization pattern on the defined problem space was investigated, and the results obtained were graphed for comparative presentation. The graphing is grouped based on the dataset used for experimentation so that each graph presents a comparative outline of curves for some selected algorithms considered in this study. Values used for the graphing were the fitness value over all iterations in each case of the datasets on all optimization algorithms. Again, these plots illustrate the convergence pattern for each algorithm as experimented on different datasets. The BDMO, BDMSAO, GNDO-SA, ASGW, HSGW, AIEOU, WOA, RSGW, RTHS, and PSO algorithms were considered in the convergence plots.

Figure 2 shows the convergence plots for ionosphere, congressEW, Exactly, Exactly2, and Vote datasets. For the ionosphere dataset, curves of GNDO-SA, ASGW, HSGW, AIEOU, WOA, RSGW, RTHS, and PSO run below those of BDMO and BDMSAO. A similar pattern is repeated for congressEW, Exactly, and Exactly2 datasets. Interestingly, for the Vote dataset, the PSO algorithms perform better than all algorithms with the BDMO algorithm. A competitive performance seen with both BDMO and BDMSAO overlap in the congressEW and Exactly2 datasets but shows the slight distance in the case of the ionosphere, Exactly, and Vote datasets. The highest fitness value of 1.0 obtained for all 50 iterations is reported by PSO in the Vote dataset. The relative highest values obtained for 0.9 and above were those seen in BDMO and BDMSAO on the ionosphere, congressEW, and Vote datasets. The performance of BDMO and BDMSAO algorithms demonstrates a superior performance when compared with all the similar methods in all the five datasets compared in the figure. This shows that the algorithms are suitable for finding the minimum number of features required for classifying class distributions in the datasets.

**Figure 3:**
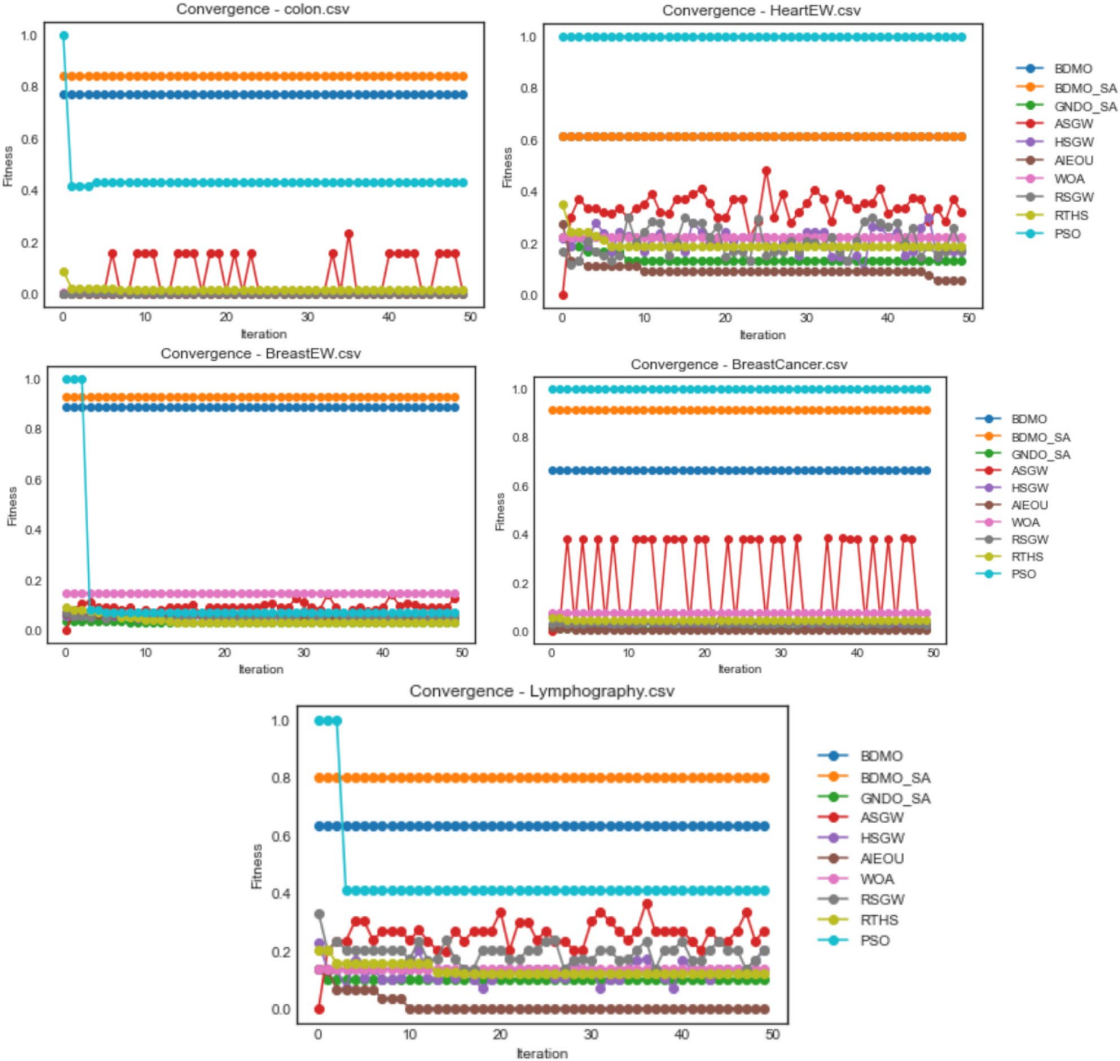
Convergence behavior of all algorithms on colon, HeartEW, BreastEW, BreastCancer, and Lymphography datasets.

In Fig. 3, the convergence curve for the ASGW algorithm for all datasets of the colon, HeartEW, BreastEW, BreastCancer, and Lymphography are seen to spike with a measure of instability from the first iteration to the last. Those for GNDO-SA, HSGW, RTHS, RSGW, WOA, and AEIOU are poorly fitted, considering the convergence curves for these algorithms lying far below those of PSO, BDMO, and BDMO-SA. Note that in all datasets listed in the figure, PSO, BDMO, and BDMO-SA are seen to competitively converge above all other methods confirming the superiority of the three methods when compared with others. However, in all the five datasets reported in the figure, BDMO and BDMO-SA performed better than PSO, which only converges based on its function evaluation values above the other two when HeartEW and BreastCancer datasets were experimented with. In all cases where BDMO and BDMO-SA performed well above others, we see the fitness values obtained lined through values above 0.8. This significant classification value confirms that the number of features selected for the two algorithms in those datasets represents a high-quality selection.

**Figure 4:**
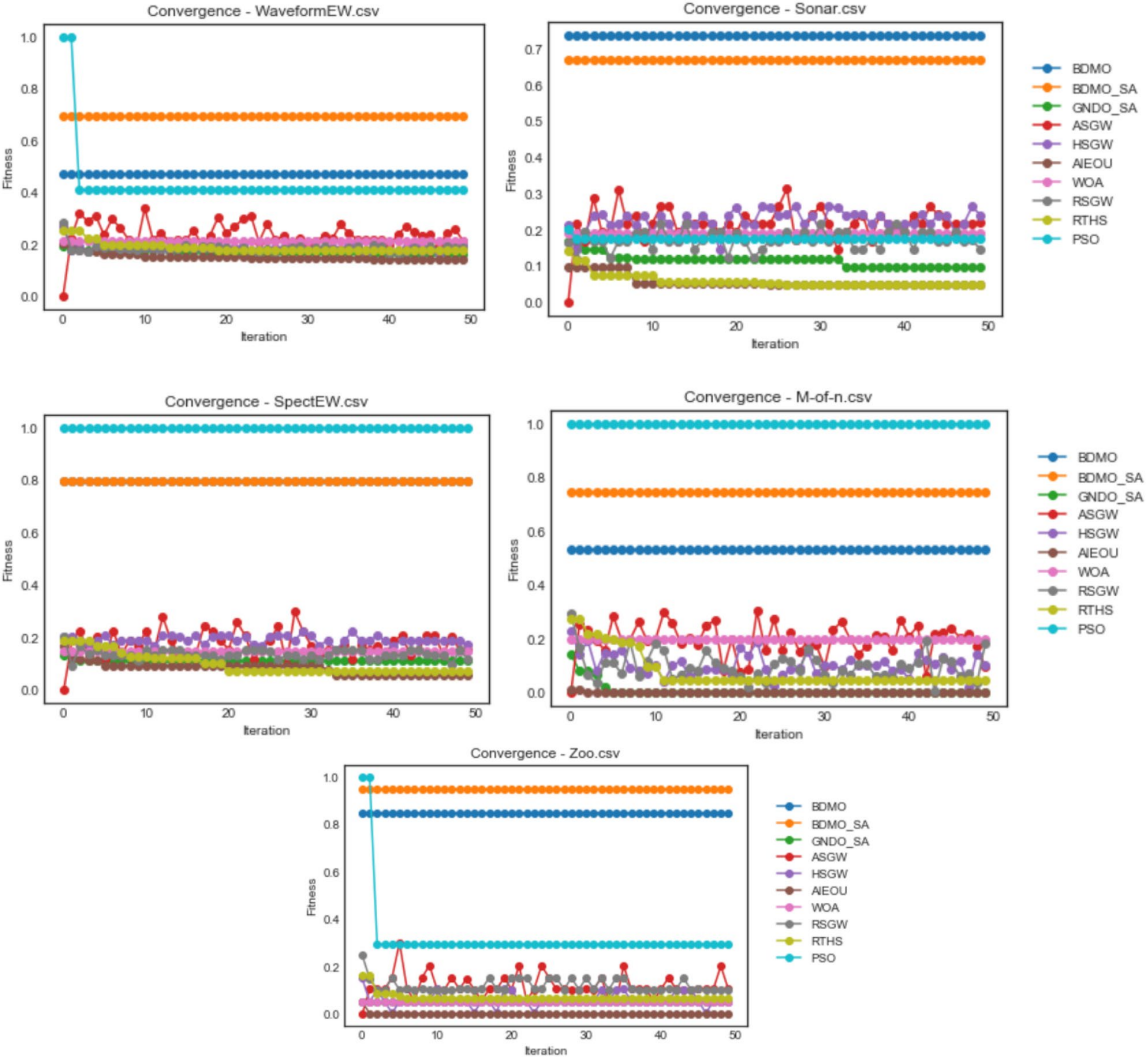
Convergence behavior of all algorithms on waveformEW, sonar, SpaceEW, M-of-n, and Zoo datasets.

The BDMO and BDMO-SA are reported to have performed well in three of the five datasets used for the plots in Fig. 4. Although the PSO algorithm showed a competitive performance with the two algorithms, we note that these are only limited to the SpaceEW and M-of-n datasets. Meanwhile, other algorithms have their curves running below those for PSO, BDMO and BDMO-SA in all the five datasets. We discovered that the nature of the five datasets observed in the figure is computationally demanding, given the unstable performance of the ASGW and RSGW.

**Figure 5:**
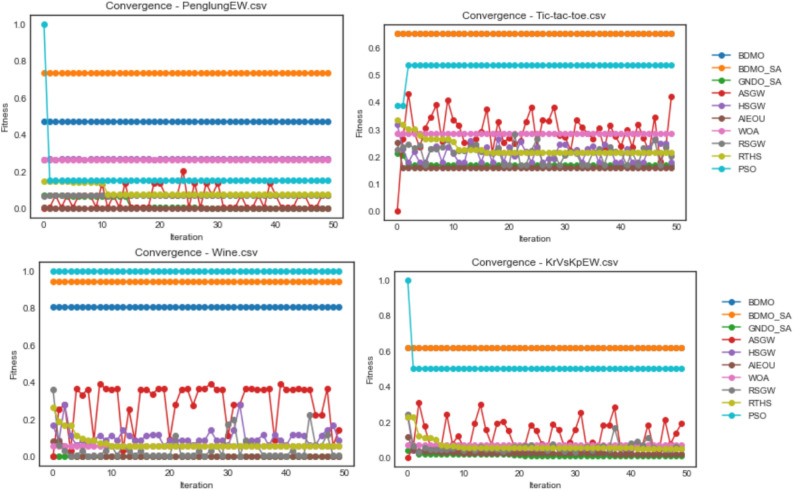
Convergence behavior of all algorithms on PenglungEW, Tic-tac-toe, Wine, and KrVsKpEW datasets.

Figure 5 shows the convergence curves for all algorithms on PenglungEW, Tic-tac-toe, Wine, and KrVsKpEW datasets. Interestingly, the impact of hybridizing BDMO with the SA algorithm proved outstanding as the algorithm competes with PSO well in three cases. The computational difficulty experienced with KrVsKpEW for all algorithms still puts the BDMO-SA algorithm ahead of others to demonstrate that the proposed hybrid algorithm is suitable for selecting the optimal number of features required for solving the classification problem. These outstanding performances are not limited to the KrVsKpEW dataset alone but span across all datasets considered in this study.

**Figure 6:**
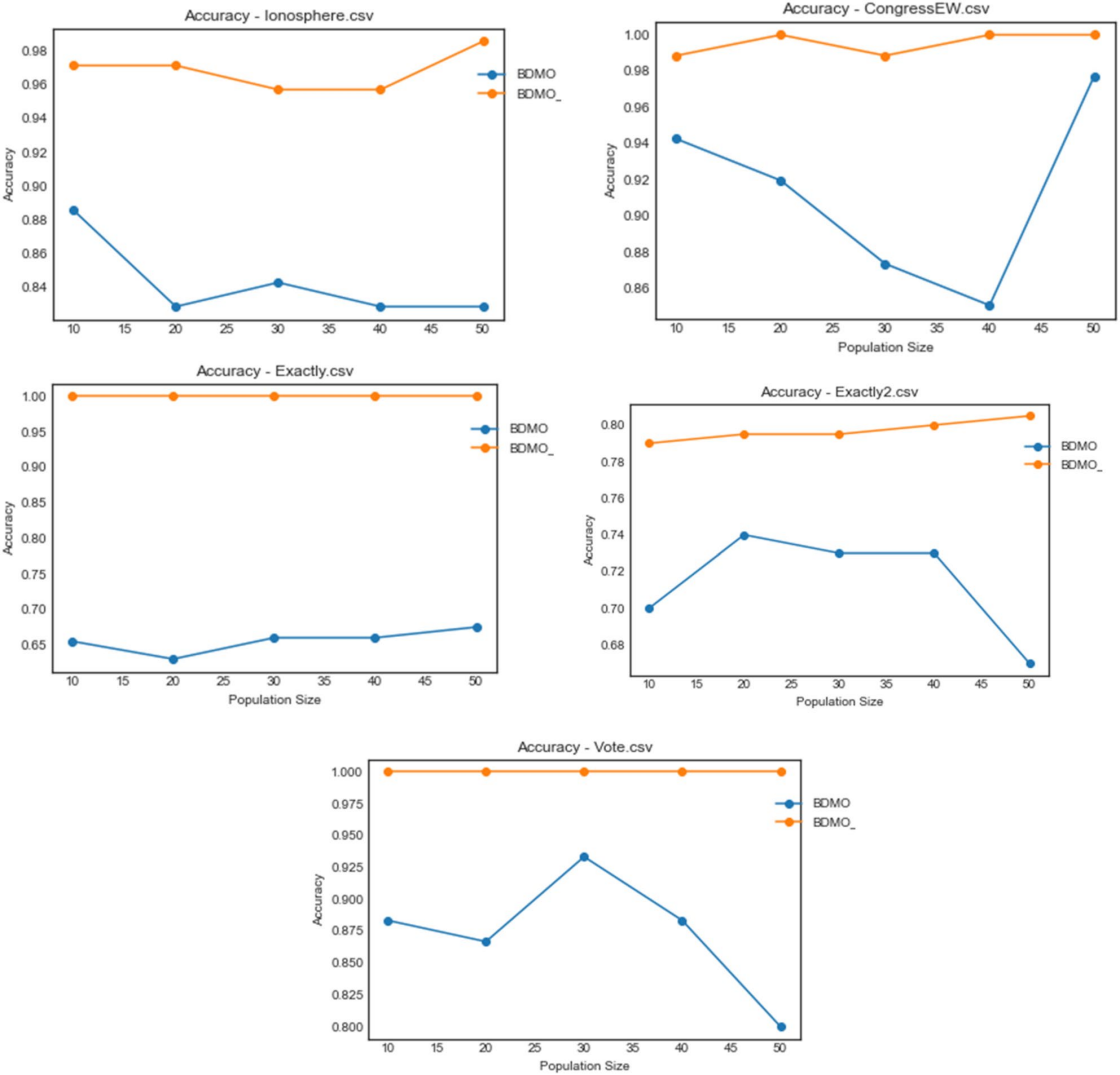
Accuracy plot of the BDMSAO and BDMO on the ionosphere, congressEW, Exactly, Exactly2, and Vote datasets.

**Figure 7:**
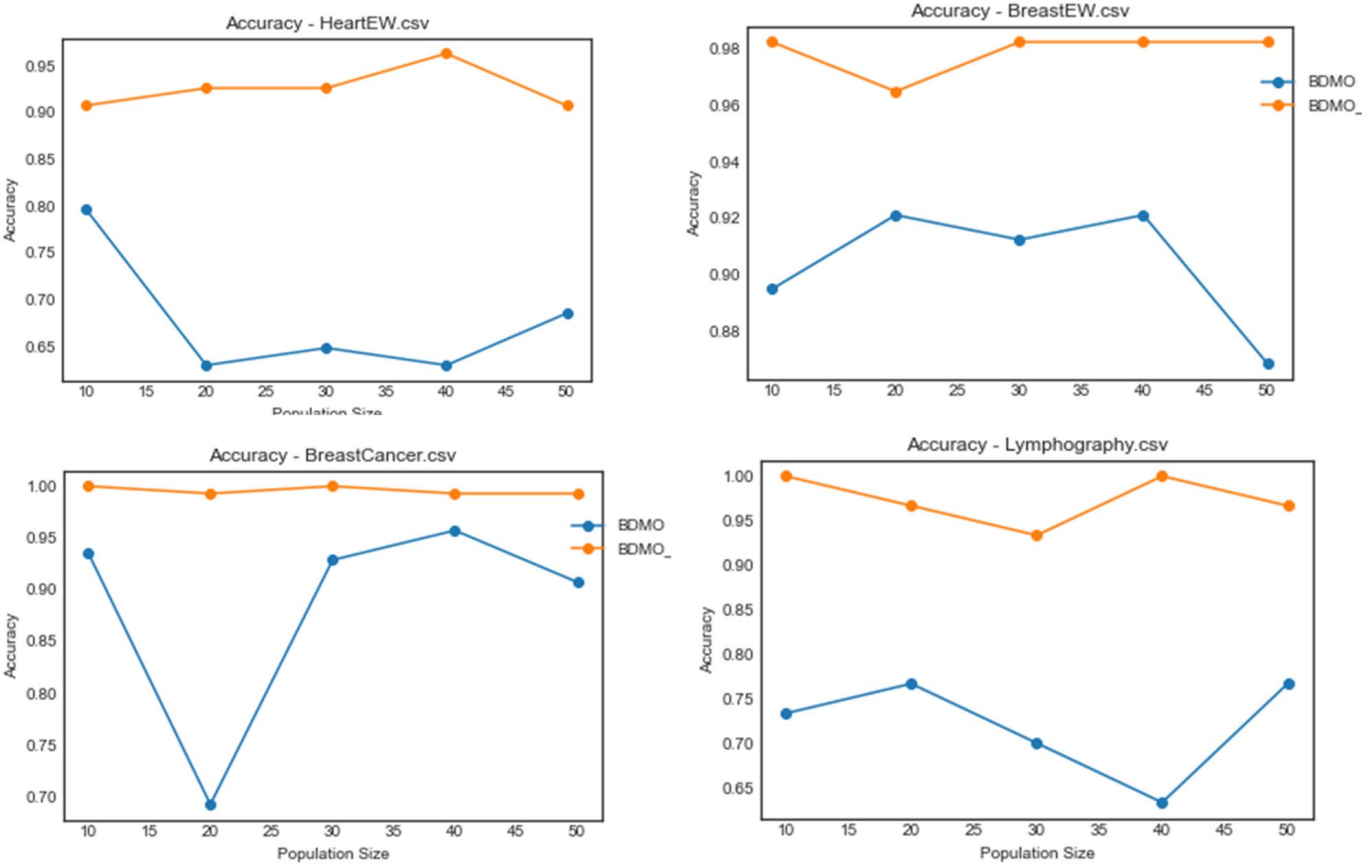
Accuracy plot of the BDMSAO and BDMO on HeartEW, BreastEW, BreastCancer, and Lymphography datasets.

**Figure 8:**
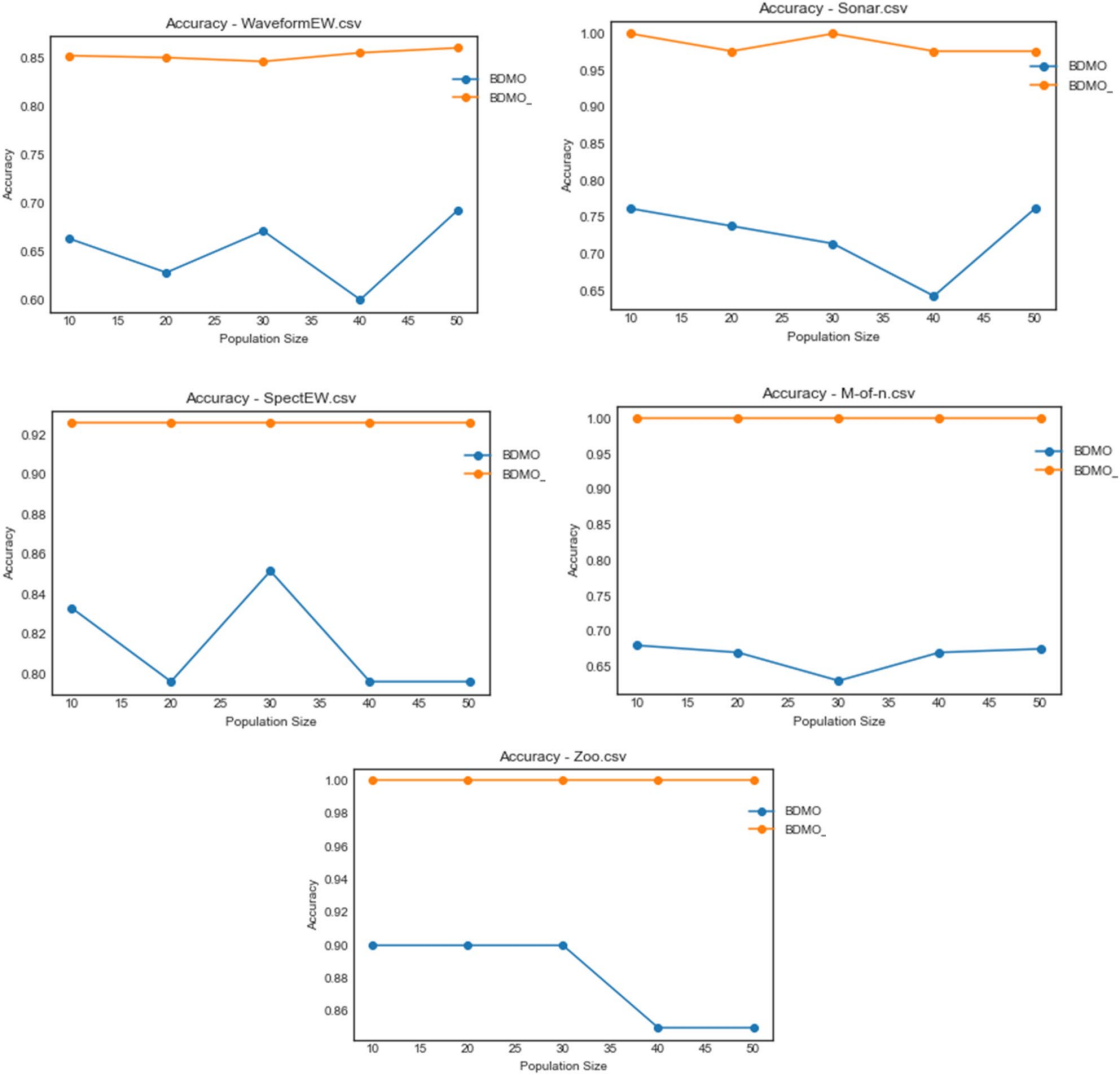
Accuracy plot of the BDMSAO and BDMO on waveformEW, sonar, SpaceEW, M-of-n, and Zoo datasets.

**Figure 9:**
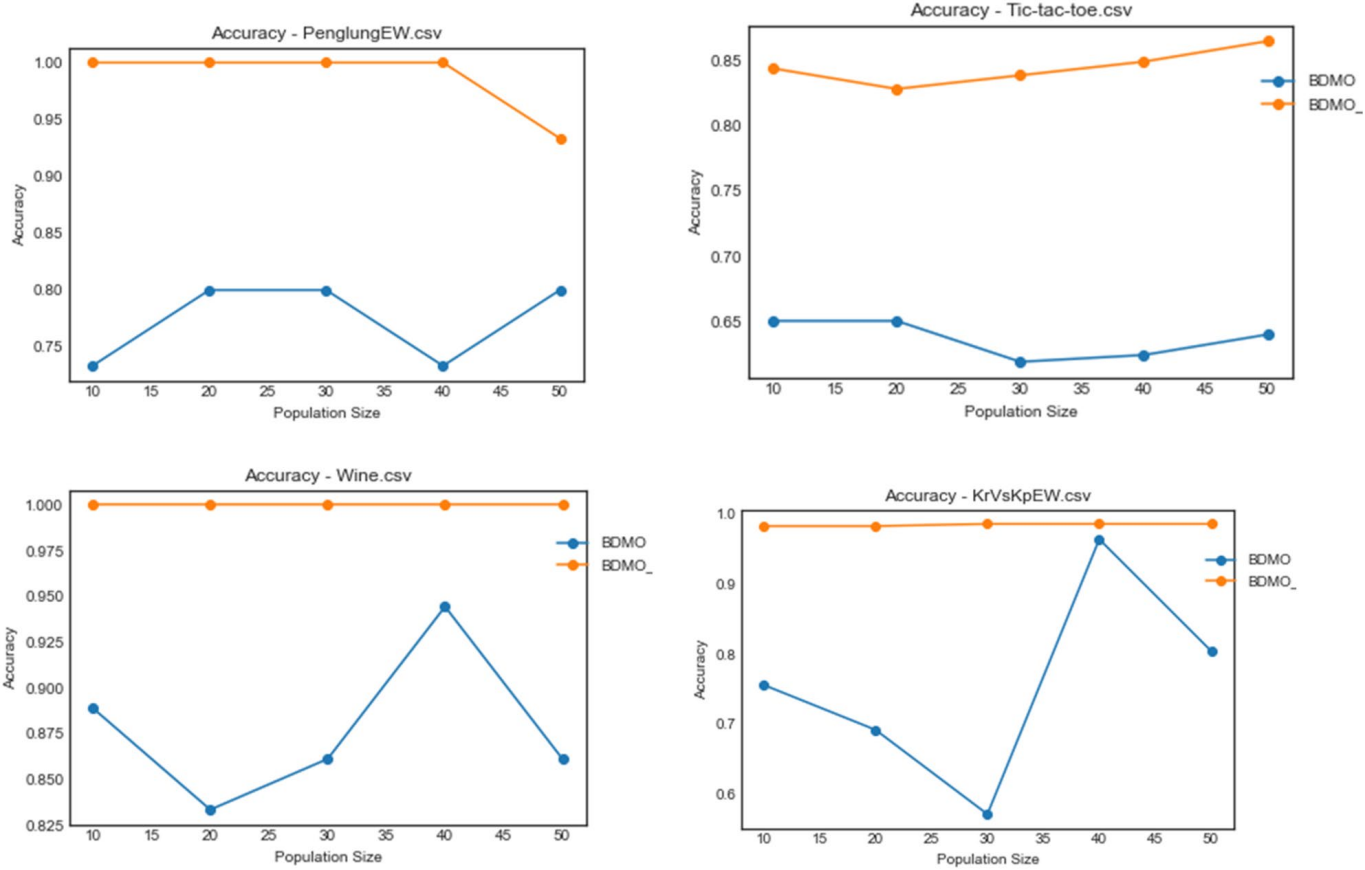
Accuracy plot of the BDMSAO and BDMO on PenglungEW, Tic-tac-toe, Wine, and KrVsKpEW datasets.

Considering the outstanding and competitive performance of the BDMSAO and BDMO algorithms, as reported in previous paragraphs, the plots for accuracy are concentrated only on the two algorithms for convenient comparative analysis. In Figs. 6, 7, 8 and 9. A plot for accuracy against iteration for all datasets is presented for BDMSAO and BDMO. In Fig. 6, the accuracy obtained for BDMSAO for all the five datasets indicates that it performs better than the base algorithm, which is the BDMO. Where we see the curve for BDMSAO rising, that of BDMO was dropping to rise in experimentation with some datasets. Meanwhile, all accuracies for the datasets considered rose above 0.9, with those of Exactly and Vote running on 1.0 accuracies for all iterations. Figure 7 shows the curve for accuracies obtained for HeartEW, BreastEW, BreastCancer, and Lymphography datasets over all iterations. Similar to the previous discussion, we see the hybrid method of BDMSAO doing well in all datasets when compared with BDMO.

The plot for the accuracy values obtained for the datasets on waveformEW, sonar, SpaceEW, M-of-n, and Zoo are shown in Fig. 8. There is a significant difference in the performance of BDMSAO when compared with its corresponding base algorithm. In most cases, the values for accuracy obtained were rising to 1.0, whereas those of BDMO were lying below 0.8. Again, this result demonstrates that the accuracy of the features selection result discussed in previous sections is consistent and represents an outstanding performance of the new hybrid method. Figure 9 shows a comparison of the curve for PenglungEW, Tic-tac-toe, Wine, and KrVsKpEW datasets. The BDMO performance in these datasets rose above what has been observed in other datasets, though it still lags behind that of the hybrid method, the BDMSAO proposed and implemented in this study.

### Comparison

The discussion earlier clarifies that the BDMSAO outperforms the BDMO. In this subsection, the performance of the proposed hybrid method is compared with nine other state-of-the-art methods, out of which seven are hybrid methods. The algorithms that were compared with other methods like adaptive switching grey-whale optimizer (ASGW), social ski driver algorithm and late acceptance hill-climbing (SSDs + LAHC)^83^, serial grey-whale optimizer (HSGW), embedded chaotic whale survival algorithm (ECWSA-4)^84^, binary GA, random switching grey-whale optimizer (RSGW), electrical harmony-based metaheuristic (EHHM), BPSO, and binary simulated normal distribution optimizer (BSNDO)^1^.

Given the results obtained in Table 5, we can conclude that the BDMSAO and BSNDO yield better results than other methods on 11 out of 18 datasets, which is 61.11%. Meanwhile, the BDMSAO produced a 100% accuracy on 9 out of 18 datasets (50%), while its competitor, the BSNDO, produced 100% accuracy on 8 out of 18 datasets (44.4%). On the Breastcancer dataset, the BDMSAO, BSNDO, and EHHM achieved 100% accuracy. The proposed BDMSAO produced the second-best result and BSNDO and SSDs + LAHC on the BreastEW dataset after ASGW and EHHM. On the CongressEW dataset, BDMSAO and EHHM came third after BSNDO & SSDs + LAHC and ASGW. Most of the methods in this study, including the BDMSAO, yield 100% accuracy on the Exactly dataset. On the Exactly2 dataset, the BDMSAO achieved the third best result after BSNDO and EHHM, which narrowly beat the BDMSAO by a 0.1% margin. The BDMSAO & BSNDO produced the third best result on HeartEW after HSGW & SSDs + LAHC. On IonosphereEW, BDMSAO also generated the third best result after EHHM & ASGW, which narrowly beat our method by 0.06%. The BDMSAO yielded the best result of 98.75% on the KrvskpEW dataset. On Lymphography and Sonar datasets, the BDMSAO yielded 100% and 97.62% accuracy on SpecEW to lead other methods. On M-of-n, PenguinEW, Wine, and Zoo datasets, the BDMSAO achieved 100% accuracy with other methods. Finally, the BDMSAO achieved the third best result on WaveformEW and produced the fifth best in Tic-tac-toe.

**Table 5:** Classification accuracy of BDMSAO in comparison with other well-known feature selection methods tested using the UCI dataset, and the highlighted results indicate the highest classification accuracy.

No	Datasets	BDMSAO	ASGW	BSNDO	HSGW	BPSO	BGA	RSGW	SSDs + LAHC	ECWSA-4	EHHM
1	Breastcancer	**100**	98.5	**100**	98.6	96.29	97.43	97.1	98.93	95.21	**100**
2	BreastEW	98.25	**100**	98.25	98.1	97.19	97.54	98.2	98.25	97.38	**100**
3	CongressEW	98.85	99.4	**100**	97.5	96.33	96.79	96.1	**100**	96.23	98.85
4	Exactly	**100**	99.9	**100**	**100**	**100**	**100**	99.7	**100**	78.09	**100**
5	Exactly2	79	77.7	**80.5**	81.5	76.8	77	77.9	79	78.9	79.1
6	HeartEW	90.74	83.1	90.74	**92.3**	83.7	87.41	84.8	91.67	85.63	90.7
7	IonosphereEW	97.14	97.2	95.74	94.4	94.89	94.89	97.8	96.43	86.79	**98.6**
8	KrvskpEW	**98.75**	97.1	98.44	97.3	98.31	98.5	97.2	97.81	93.53	97.81
9	Lymphography	**100**	88.4	96.67	93.4	89.19	83.78	89.3	96.67	87.02	96.9
10	M-of-n	**100**	**100**	**100**	**100**	**100**	**100**	**100**	**100**	92.47	**100**
11	PengiunEW	**100**	**100**	**100**	94.2	91.89	91.89	**100**	**100**	87.63	**100**
12	SonarEW	**100**	94.8	95.24	96.4	94.23	**99.04**	97.9	97.62	76.84	92.85
13	SpectEW	**97.62**	87	96.22	86.2	88.81	89.55	81.5	95.15	79.84	90.74
14	Tic-tac-toe	84.38	86.5	**87.5**	82.8	79.96	79.96	85.9	87.24	78.75	85
15	Vote	**100**	98.4	**100**	98.3	96	97.33	99.6	**100**	95.08	98.4
16	WaveformEW	85.20	74.6	**87**	74.8	75.6	78.36	75.7	84.4	80.18	86.8
17	Wine	**100**	**100**	**100**	**100**	97.75	98.88	**100**	**100**	98.02	**100**
18	Zoo	**100**	**100**	**100**	**100**	96.08	90.2	**100**	**100**	98.95	**100**

Table 6 shows the performance of the BDMSAO and other feature selection methods used in this study regarding the number of features selected. This proposed approach selected the least number of features on the Tic-tac-toe dataset. It also selected the least features on the CongressEW dataset along with BPSO and the Vote dataset with BPSO & BSNDO. It performed second in selecting the least features on Breastcancer, BreastEW, Exactly, HeartEW, and M-of-n datasets, while it performed third on Exactly2 datasets. It attained the fourth best results on Lymphography, WaveformEW, Wine, & Zoo, and achieved fifth on IonosphereEW, KrvskpEW, PenguinEW, and SonarEW datasets.

**Table 6:** The number of features selected by BDMSAO compared with other well-known feature selection methods tested using the UCI dataset. The highlighted results indicate the least number of feature subsets selected.

No	Datasets	BDMSAO	ASGW	BSNDO	HSGW	BPSO	RSGW	SSDs + LAHC	ECWSA-4)	EHHM
1	Breastcancer	4	4.867	4	5	4	5.933	**2.5**	4	7
2	BreastEW	7	15.833	**4**	16.667	9	17.5	9	13	15
3	CongressEW	**3**	8.833	7	8.867	**3**	9.7	5.5	7	5
4	Exactly	6	6.867	6	6.7	6	7.1	6	7	7
5	Exactly2	6	7.933	8	9.033	**1**	9.2	8	5	9
6	HeartEW	4	6.367	4	8.767	**3**	6.133	5	8	9
7	IonosphereEW	19	17.3	16	18.167	**7**	20.5	12	**7**	10
8	KrvskpEW	19	24.5	22	24.8	12	24.8	20	15	16
9	Lymphography	10	11.2	**5**	10.567	**5**	10.567	6.5	6	10
10	M-of-n	6	6.867	6	6.8	6	7.1	6	7	**5**
11	PengiunEW	132	170.3	187	135.33	130	181.2	140	**74**	93
12	SonarEW	26	35.5	27	34.3	22	36.433	23.5	22	23
13	SpectEW	22	10.167	6	10.233	6	13.3	9	11	7
14	Tic-tac-toe	**4**	7	8	7	6	7	9	6	8
15	Vote	**3**	8.967	**3**	7.567	**3**	8.8	4.5	5	6
16	WaveformEW	24	25.833	33	26.933	**15**	27.533	22.5	20	**15**
17	Wine	4	5.933	3	4.533	5	5.867	3	**1**	7
18	Zoo	5	7.6	5	5.533	5	5.3	4.5	**1**	7

### Statistical test

In Table 7, the Friedman mean ranking test results are shown in bold values as the best-ranked algorithm. In most cases, the BPSO ranked highest, as seen in the table, and our proposed method ranked second in all cases where the BPSO ranked first. However, the BDMSAO ranked better than the BPSO on Exactly and Exactly2 datasets and then tied with the BPSO on two datasets i.e. PenguinEW and Sonar. It also outranks other methods (that are hybrid methods as our proposed method) in the ranking. This statistically shows the performance significance of the BDMSAO over other algorithms.

**Table 7:** Friedman mean ranking test.

No	Datasets	BDMSAO	ASGW	BSNDO	HSGW	BPSO	RSGW
1	Breastcancer	5.00	1.00	2.30	3.50	**5.90**	3.20
2	BreastEW	5.40	1.00	2.50	3.30	**5.50**	3.30
3	CongressEW	5.40	1.00	2.20	3.10	**5.60**	3.70
4	Exactly	**5.40**	1.00	2.00	3.85	5.30	3.45
5	Exactly2	**4.50**	1.00	2.10	6.00	4.40	3.00
6	HeartEW	5.30	1.00	2.20	3.20	**5.70**	3.60
7	IonosphereEW	5.20	1.00	2.40	3.60	**5.70**	3.10
8	KrvskpEW	5.00	1.00	2.30	3.40	**5.90**	3.40
9	Lymphography	5.10	1.00	2.10	3.80	**5.70**	3.30
10	M-of-n	5.00	1.00	2.00	3.30	**6.00**	3.70
11	PengiunEW	**5.50**	1.00	3.05	3.00	**5.50**	2.95
12	SonarEW	**5.40**	1.00	2.35	3.35	**5.40**	3.50
13	SpectEW	5.20	1.00	2.30	3.40	**5.80**	3.30
14	Tic-tac-toe	5.10	1.00	2.30	3.70	**5.60**	3.30
15	Vote	5.10	1.00	2.80	3.10	**5.90**	3.10
16	WaveformEW	5.10	1.00	2.00	3.40	**5.90**	3.60
17	Wine	5.00	1.00	3.00	3.00	**5.70**	3.30
18	Zoo	5.10	1.00	2.15	3.65	**5.90**	3.20

The statistical significance of the BDMSAO and other algorithms are the same on average on all measures on most of the datasets used, producing significant values less than 0.05. The 0.05 is the representation of the significant level of 5%, which is used in the acceptance of the null value. As our proposed method generated more values that are less than 0.05 than all other algorithms in all datasets except for the BPSO. Therefore, this validates the fact that the samples were produced from a continuous distribution having the same medians as opposed to the null hypothesis that does not. This gives convincing proof that the results obtained by the BDMSAO are statistically significant compared to other similar methods. Table 8 presents the Wilcoxon mean rank test.

**Table 8:** Wilcoxon mean rank test.

No	Datasets	ASGW-BDMSAO	BSNDO- BDMSAO	HSGW- BDMSAO	BPSO- BDMSAO	RSGW- BDMSAO
1	Breastcancer	0.005	0.005	0.005	0.074	0.005
2	BreastEW	0.005	0.005	0.005	0.058	0.005
3	CongressEW	0.005	0.005	0.005	0.508	0.005
4	Exactly	0.005	0.005	0.005	0.169	0.005
5	Exactly2	0.005	0.005	0.005	0.202	0.005
6	HeartEW	0.005	0.005	0.005	0.047	0.005
7	IonosphereEW	0.005	0.005	0.005	0.386	0.005
8	KrvskpEW	0.005	0.007	0.005	0.007	0.005
9	Lymphography	0.005	0.005	0.005	0.074	0.005
10	M-of-n	0.005	0.005	0.005	0.005	0.005
11	PengiunEW	0.005	0.005	0.005	0.645	0.005
12	SonarEW	0.005	0.005	0.005	0.508	0.005
13	SpectEW	0.005	0.005	0.005	0.386	0.005
14	Tic-tac-toe	0.005	0.009	0.005	0.049	0.005
15	Vote	0.005	0.005	0.005	0.074	0.005
16	WaveformEW	0.005	0.005	0.005	0.009	0.005
17	Wine	0.005	0.007	0.005	0.285	0.007
18	Zoo	0.005	0.005	0.005	0.007	0.005

## Testing on high-dimensional datasets

**Figure 10:**
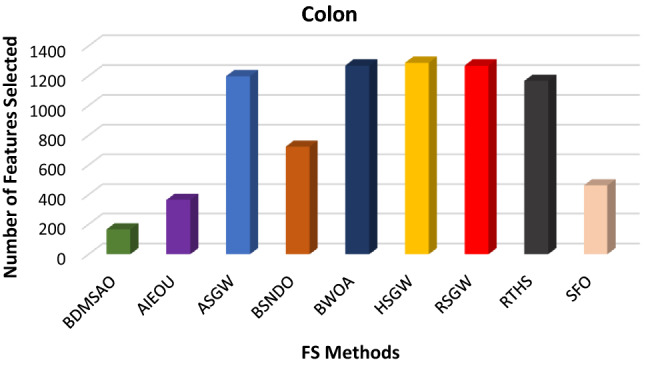
The number of features selected by BDMSAO and other FS methods on the Colon dataset.

**Figure 11:**
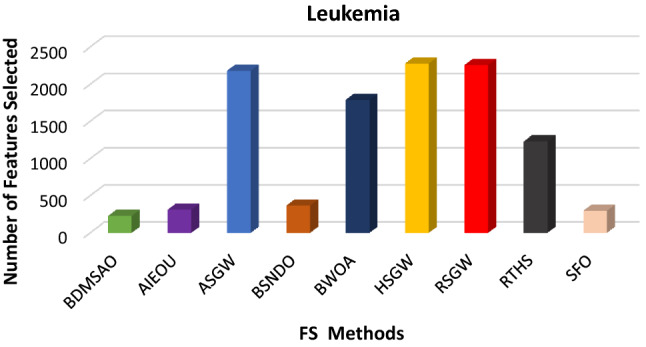
The number of features selected by BDMSAO and other FS methods on Leukemia.

**Figure 12:**
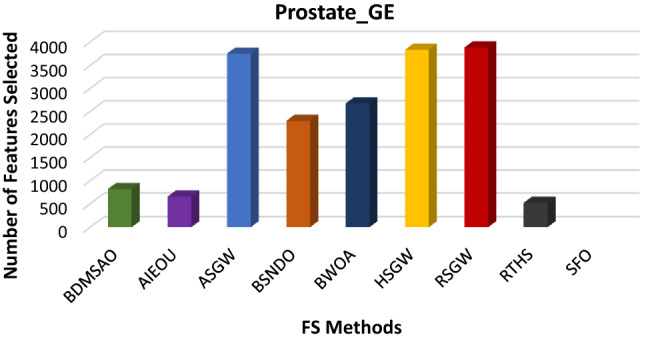
The number of features selected by BDMSAO and other FS methods on the Prostate_GE dataset.

The results discussed above revealed the performance of the BDMSAO over other well-known algorithms used in this study. To evaluate the robustness of this proposed algorithm, we tested its application to three high-dimensional datasets known to be extremely challenging. The dataset's description is provided in Table 9. The efficacy of the BDMSAO is also proven in comparison with eight (8) well-known state-of-the-art feature selection methods mentioned in Table 10. The number of features selected by BDMSAO compared with some popular FS selection methods is shown in Figs. 10, 11, and 12. All algorithms, including the BDMSAO, yielded the highest classification accuracy on both high-dimensional datasets. The BDMSAO selected the least number of features on the colon and leukemia datasets, respectively, compared to those achieved by the AIEOU, SFO and BSNDO, thus confirming the proposed method's ability to select the least features as indicated previously. However, the BDMSAO selected the third best number of features after RTHS and AIEOU on the Prostate_GE dataset.

**Table 9:** High-dimensional datasets and their properties.

Number	Datasets	# features	# instances	# Classes	Categories
1	Colon	2000	62	2	Biological
2	Leukemia	7070	72	2	Biological
3	Prostate_GE	5966	102	2	Biological

**Table 10:** Classification accuracy produced by BDMSAO and other FS methods on high-dimensional datasets.

Datasets	BDMSAO	AIEOU	ASGW	BSNDO	BWOA	HSGW	RSGW	RTHS	SFO
Colon	100	100	100	100	100	100	100	100	100
Leukemia	100	100	100	100	100	100	100	100	100
Prostate_GE	100	100	100	100	100	100	100	100	NA

## Conclusion and future work

This paper proposed a new hybridized feature selection problem-solving method called the BDMSAO. The hybridization concept emanates from the methodological enhancement of the standard BDMO and SA algorithms. The developed BDMSAO utilized the SA as a local search method to enhance the exploitation of the BDMO and aid a suitable balance between the exploitation and exploration of the hybrid method. Interestingly, the BDMSAO accomplished a substantial enhancement in solving feature selection problems regarding classification accuracy achieved against the BDMO and other well-known state-of-the-art algorithms used for comparison in this study.

The performances of the proposed approaches were assessed and compared against nine other feature selection methods, including the ASGW, BSNDO, HSGW, BPSO, BGA, RSGW, SSDs + LAHC, ECWSA-4, and EHHM, respectively. The evaluation criteria reported for each approach include the classification accuracy, average feature selection size number, and the respective algorithms' convergence characteristics. Similarly, the BDMSAO was compared against the BDMO algorithm to ascertain the validity of the initial enhancement claim over the BDMO.

This developed feature selection approach was mainly evaluated and validated on some UCI datasets confirmed to be challenging. The new method was also tested using three high-dimensional datasets to prove its robustness in finding reasonable solutions to real-world problems that are often considered complex and difficult to solve using conventional methods. The results obtained by the BDMSAO indicate that the proposed method is applicable in various publicly available datasets. A limitation of this study may be in the computation complexity due to the addition of a local search technique.

In the future, it would be interesting to consider hybridizing the BDMO with other state-of-the-art metaheuristics such as the GA, PSO, CSO, GWO, PDO and KHA algorithms. Also, it will be worth considering employing the hybrid BDMSAO algorithm in other real-world problem areas like image processing, facial recognition, and text classification, where researchers utilize a huge dimensional vector of features without knowing the reputation of every dataset features.

## Data Availability

All data generated or analyzed during this study are included in this published article.

## References

[1] Ahmed S, Sheikh KH, Mirjalili S, Sarkar R (2022). Binary simulated normal distribution optimizer for feature selection: Theory and application in COVID-19 datasets. Expert Syst. Appl..

[2] Dash M, Liu H (1997). Feature selection for classification. Intell. Data Anal..

[3] Guyon I, Elisseeff A (2003). An introduction to variable and feature selection. J. Mach. Learn. Res..

[4] He X, Cai D, Niyogi P (2005). Laplacian score for feature selection. Adv. Neural Inf. Process. Syst..

[5] Liu H, Motoda H (2012). Feature Selection for Knowledge Discovery and Data Mining.

[6] Li Y, Li T, Liu H (2017). Recent advances in feature selection and its applications. Knowl. Inf. Syst..

[7] Li J, Cheng K, Wang S, Morstatter F, Trevino RP, Tang J, Liu H (2017). Feature selection: A data perspective. ACM Comput. Surv..

[8] Chhikara RR, Sharma P, Singh L (2018). An improved dynamic discrete firefly algorithm for blind image steganalysis. Int. J. Mach. Learn. Cybern..

[9] Sesmero MP, Alonso-Weber JM, Gutierrez G, Ledezma A, Sanchis A (2015). An ensemble approach of dual base learners for multi-class classification problems. Inf. Fusion.

[10] Vaiyapuri T, Alaskar H, Aljohani E, Shridevi S (2022). Red fox optimizer with data-science-enabled microarray. Appl. Sci..

[11] Xue B, Zhang M, Browne WN (2014). Particle swarm optimization for feature selection in classification: Novel initialization and updating mechanisms. Appl. Soft Comput. J..

[12] Žerovnik J (2015). Heuristics for NP-hard optimization problems - simpler is better!?. Logist. Sustain. Transp..

[13] Faris H, Hassonah MA, Al-Zoubi AM, Mirjalili S, Aljarah I (2018). A multi-verse optimizer approach for feature selection and optimizing SVM parameters based on a robust system architecture. Neural Comput. Appl..

[14] Hammouri AI, Mafarja M, Al-Betar MA, Awadallah MA, Abu-Doush I (2020). An improved Dragonfly Algorithm for feature selection. Knowl.-Based Syst..

[15] Lai C, Reinders MJT, Wessels L (2006). Random subspace method for multivariate feature selection. Pattern Recogn. Lett..

[16] Talbi EG (2009). Metaheuristics: From Design to Implementation.

[17] Kennedy, J. & Eberhart, R. Particle swarm optimization. In *Proceedings of ICNN'95-International Conference on Neural Networks*, Vol. 4, 1942–1948. IEEE (1995).

[18] Davis L (1991). Handbook of Genetic Algorithms.

[19] Karaboga D, Basturk B (2007). A powerful and efficient algorithm for numerical function optimization: Artificial bee colony (ABC) algorithm. J. Glob. Optim..

[20] Mirjalili S (2016). SCA: A Sine Cosine Algorithm for solving optimization problems. Knowl.-Based Syst..

[21] Yang X-S, Deb S (2009). Cuckoo search via l´evy flights. IEEE World Congr..

[22] Geem ZW, Kim JH, Loganathan GV (2001). A new heuristic optimization algorithm: Harmony search. Simulation.

[23] Mirjalili SM, Mirjalili SM, Lewis A (2014). Grey Wolf optimizer. Adv. Eng. Softw..

[24] Wang G, Guo L, Gandomi AH, Cao L, Alavi AH, Duan H, Li J (2013). Lévy-flight krill herd algorithm. Math. Probl. Eng..

[25] Ezugwu AE, Agushaka JO, Abualigah L, Mirjalili S, Gandomi AH (2022). Prairie Dog Optimization Algorithm. Neural Comput. Appl..

[26] Remeseiro B, Bolon-Canedo V (2019). A review of feature selection methods in medical applications. Comput. Biol. Med..

[27] Wolpert DH, Macready WG (1997). No free lunch theorems for optimization. IEEE Trans. Evol. Comput..

[28] Almugren N, Alshamlan H (2019). A survey on hybrid feature selection methods in microarray gene expression data for cancer classification. IEEE Access.

[29] Talbi E-G (2002). A taxonomy of hybrid metaheuristics. J. Heuristics.

[30] Kirkpatrick S, Gelatt CD, Vecchi MP (1983). Optimization by simulated annealing. Science.

[31] Bhattacharyya T, Chatterjee B, Singh PK, Yoon JH, Geem ZW, Sarkar R (2020). Mayfly in harmony: A new hybrid meta-heuristic feature selection algorithm. IEEE Access.

[32] Sheikh KH, Ahmed S, Mukhopadhyay K, Singh PK, Yoon JH, Geem ZW, Sarkar R (2020). EHHM: Electrical harmony based hybrid meta-heuristic for feature selection. IEEE Access.

[33] Gendreau M, Potvin J-Y (2005). Metaheuristics in Combinatorial Optimization. Ann. Oper. Res..

[34] Abdel-Basset M, Abdel-Fatah L, Sangaiah AK, Sangaiah AK, Sheng M (2018). Chapter 10: Metaheuristic algorithms: A comprehensive review. Intelligent Data-Centric Systems.

[35] Oh I-S, Lee J-S, Moon B-R (2004). Hybrid genetic algorithms for feature selection. IEEE Trans. Pattern Anal. Mach. Intell..

[36] Mirjalili S, Lewis A (2016). The whale optimization algorithm. Adv. Eng. Softw..

[37] Agushaka JO, Ezugwu AE, Abualigah L (2022). ScienceDirect Dwarf Mongoose Optimization Algorithm. Comput. Methods Appl. Mech. Eng..

[38] Agushaka JO, Ezugwu AE, Abualigah L (2021). Gazelle Optimization Algorithm: A novel nature-inspired metaheuristic optimizer for mechanical engineering applications. PLoS ONE.

[39] Moradi P, Gholampour M (2016). A hybrid particle swarm optimization for feature subset selection by integrating a novel local search strategy. Appl. Soft Comput. J..

[40] Talbi E, Jourdan L, Garcia-Nieto J, Alba E (2008). Comparison of population based metaheuristics for feature selection: Application to microarray data classification. IEEE/ACS Int. Conf. Comput. Syst. Appl..

[41] Yong Z, Dun-wei G, Wan-qiu Z (2016). Feature selection of unreliable data using an improved multi-objective PSO algorithm. Neurocomputing.

[42] Jona JB, Nagaveni N (2012). A hybrid swarm optimization approach for feature set reduction in digital mammograms. WSEAS Trans. Inf. Sci. Appl..

[43] Babatunde RS, Venkat I, Babatunde RS (2014). Towards an improved face recognition system through dimensionality reduction feature dimensionality reduction using a dual level metaheuristic algorithm. Int. J. Appl. Inf. Syst..

[44] Basiri ME, Nemati S (2009). A novel hybrid ACO-GA algorithm for text feature selection. IEEE Congr. Evol. Comput..

[45] Jona J, Nagaveni N (2014). Ant-cuckoo colony optimization for feature selection in digital mammogram. Pak. J. Biol. Sci..

[46] ZorarpacI E, Özel SA (2016). A hybrid approach of differential evolution and artificial bee colony for feature selection. Expert Syst. Appl..

[47] Koza JR (1994). Genetic programming as a means for programming computers by natural selection. Stat. Comput..

[48] Glover F, Laguna M, Du D-Z, Pardalos PM (1998). Tabu Search BT: Handbook of Combinatorial Optimization: Volume1–3.

[49] Yang XS, Karamanoglu M, He X (2014). Flower pollination algorithm: A novel approach for multiobjective optimization. Eng. Optim..

[50] Moscato P, Cotta C, Mendes A (2004). Memetic Algorithms.

[51] Simon D (2008). Biogeography-based optimization. IEEE Trans. Evol. Comput..

[52] Junghans L, Darde N (2015). Hybrid single objective genetic algorithm coupled with the simulated annealing optimization method for building optimization. Energy Build..

[53] Li Y, Guo H, Wang L, Fu J (2013). A hybrid genetic-simulated annealing algorithm for the location-inventory- routing problem considering returns under E-supply chain environment. Sci. World J..

[54] Li Z, Schonfeld P (2015). Hybrid simulated annealing and genetic algorithm for optimizing arterial signal timings under oversaturated traffic conditions. J. Adv. Transp..

[55] Vasant P (2010). Hybrid simulated annealing and genetic algorithms for industrial production management problems. Int. J. Comput. Methods.

[56] Mafarja M, Abdullah S (2013). Investigating memetic algorithm in solving rough set attribute reduction. Int. J. Comput. Appl. Technol..

[57] Majdi M, Abdullah S, Jaddi NS (2015). Fuzzy population-based meta-heuristic approaches for attribute reduction in rough set theory. FADA.

[58] Azmi R, Pishgoo B, Norozi N, Koohzadi M, Baesi F (2010). A hybrid GA and SA algorithms for feature selection in recognition of hand-printed Farsi characters. IEEE Int. Conf. Intell. Comput. Intell. Syst..

[59] Manimala K, Selvi K, Ahila R (2011). Hybrid soft computing techniques for feature selection and parameter optimization in power quality data mining. Appl. Soft Comput. J..

[60] Tang, W. C. *Feature Selection For The Fuzzy Artmap Neural Network Using A Hybrid Genetic Algorithm And Tabu Search [QA76. 87. T164 2007 f rb].* http://eprints.usm.my/8145, http://eprints.usm.my/8145/1/feature_selection_for_the_fuzzy_artmap_neural_network_using_a_hybrid_genetic_algorithm_and_tabu_search.pdf. (2007).

[61] Rashedi E, Nezamabadi-pour H, Saryazdi S (2009). GSA: A gravitational search algorithm. Inf. Sci..

[62] Zhao W, Wang L, Zhang Z (2019). Atom search optimization and its application to solve a hydrogeologic parameter estimation problem. Knowl.-Based Syst..

[63] Kaveh A, Khayatazad M (2012). A new meta-heuristic method: Ray Optimization. Comput. Struct..

[64] Hosseini HS (2011). Principal components analysis by the galaxy-based search algorithm: A novel metaheuristic for continuous optimisation. Int. J. Comput. Sci. Eng..

[65] Faramarzi A, heidarinejad M, Stephens B, Mirjalili S (2020). Equilibrium optimizer: A novel optimization algorithm. Knowl.-Based Syst..

[66] Dey A, Chattopadhyay S, Singh PK, Ahmadian A, Ferrara M, Sarkar R (2020). A hybrid meta-heuristic feature selection method using golden ratio and equilibrium optimization algorithms for speech emotion recognition. IEEE Access.

[67] Ouadfel S, Abd Elaziz M (2022). Efficient high-dimension feature selection based on enhanced equilibrium optimizer. Expert Syst. Appl..

[68] Rao RV, Savsani VJ, Vakharia DP (2011). Teaching-learning-based optimization: A novel method for constrained mechanical design optimization problems. CAD Comput. Aided Des..

[69] Husseinzadeh Kashan A (2014). League Championship Algorithm (LCA): An algorithm for global optimization inspired by sport championships. Appl. Soft Comput. J..

[70] Ghorbani N, Babaei E (2014). Exchange market algorithm. Applied Soft Computing.

[71] Ramezani F, Lotfi S (2013). Social-Based Algorithm (SBA). Appl. Soft Comput. J..

[72] Dai C, Chen W, Song Y, Zhu Y (2010). Seeker optimization algorithm: A novel stochastic search algorithm for global numerical optimization. J. Syst. Eng. Electron..

[73] Sevin E, Dökeroglu T (2019). A novel hybrid teaching-learning-based optimization algorithm for the classification of data by using extreme learning machines. Turk. J. Electr. Eng. Comput. Sci..

[74] Chantar H, Tubishat M, Essgaer M, Mirjalili S (2021). Hybrid binary dragonfly algorithm with simulated annealing for feature selection. SN Comput. Sci..

[75] Elgamal ZM, Yasin NBM, Tubishat M, Alswaitti M, Mirjalili S (2020). An improved harris hawks optimization algorithm with simulated annealing for feature selection in the medical field. IEEE Access.

[76] Kurtuluş E, Yıldız AR, Sait SM, Bureerat S (2020). A novel hybrid Harris hawks-simulated annealing algorithm and RBF-based metamodel for design optimization of highway guardrails. Mater. Test..

[77] Mafarja MM, Mirjalili S (2017). Hybrid Whale Optimization Algorithm with simulated annealing for feature selection. Neurocomputing.

[78] Polap D, Woźniak M (2017). Polar bear optimization algorithm: Meta-heuristic with fast population movement and dynamic birth and death mechanism. Symmetry.

[79] Emary E, Zawbaa HM (2019). Feature selection via Lèvy Antlion optimization. Pattern Anal. Appl..

[80] Pudil P, Novovičová J, Kittler J (1994). Floating search methods in feature selection. Pattern Recogn. Lett..

[81] Song XF, Zhang Y, Guo YN, Sun XY, Wang YL (2020). Variable-size cooperative coevolutionary particle swarm optimization for feature selection on high-dimensional data. IEEE Trans. Evol. Comput..

[82] Agrawal P, Abutarboush HF, Ganesh T, Mohamed AW (2021). Metaheuristic algorithms on feature selection: A survey of one decade of research (2009–2019). IEEE Access.

[83] Chatterjee B, Bhattacharyya T, Ghosh KK, Singh PK, Geem ZW, Sarkar R (2020). Late Acceptance hill climbing based social ski driver algorithm for feature selection. IEEE Access.

[84] Guha R, Ghosh M, Mutsuddi S, Sarkar R, Mirjalili S (2020). Embedded chaotic whale survival algorithm for filter–wrapper feature selection. Soft. Comput..

